# Efferocytosis: Signaling Pathways and New Therapeutic Strategies for Diseases

**DOI:** 10.1002/mco2.70764

**Published:** 2026-05-13

**Authors:** Lei Wang, Jingjing Ge, Zehua Wang, Yihua Fang, Yongxu Jia, Ruyue Zhang, Yanru Qin

**Affiliations:** ^1^ Department of Oncology The First Affiliated Hospital of Zhengzhou University Zhengzhou China; ^2^ Department of Medical Oncology, State Key Laboratory of Oncology in South China, Guangdong Provincial Clinical Research Center for Cancer Sun Yat‐sen University Cancer Center Guangzhou China

**Keywords:** efferocytosis, immune tolerance, inflammation resolution, metabolic reprogramming, precision therapy

## Abstract

Efferocytosis—the phagocytic clearance of apoptotic cells (ACs)—is essential for maintaining tissue homeostasis, immune tolerance, and inflammation resolution. Beyond classic receptor‐mediated recognition, this process drives phagocyte metabolic reprogramming to actively facilitate tissue repair. Consequently, defective efferocytosis serves as a core pathogenic mechanism across major human diseases. This review outlines the molecular and metabolic foundations of efferocytosis and defines four universal hallmarks of its dysfunction: senescence‐driven impairment, unresolved inflammation, loss of immune tolerance, and fibrotic tissue repair. Subsequent sections explore how these defects manifest in cardiovascular, autoimmune, and neurodegenerative conditions, as well as cancer. Because efferocytosis exhibits a dual pathophysiological nature, therapeutic interventions must be highly disease‐specific. Enhancing apoptotic clearance can effectively resolve chronic inflammatory and fibrotic conditions. Conversely, because tumors hijack these same pathways to build immunosuppressive microenvironments, inhibiting efferocytosis remains a critical strategy in oncology. The synthesis of these divergent roles informs a “context‐dependent directionality” framework to guide the clinical translation of efferocytosis‐targeted precision therapies.

## Graphical abstract

1. Efferocytosis coordinates molecular signaling and metabolic reprogramming to resolve inflammation.

2. Efferocytosis dysfunction underlies four conserved pathological hallmarks across diseases.

3. Therapeutic strategies targeting efferocytosis should be context‐dependent: enhance or inhibit based on disease type.

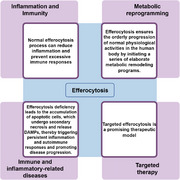

## 1 Introduction

Efferocytosis, the phagocytic clearance of apoptotic cells (ACs), serves as a critical physiological process for maintaining tissue homeostasis [1, 2, 3]. This highly coordinated process serves to sequester autoantigens and dampen proinflammatory signaling. Since its initial conceptualization, efferocytosis research has evolved from basic cell biology to a multidisciplinary field, providing crucial insights into chronic inflammation, autoimmune disorders, neurodegenerative diseases, and cancer progression. Recent studies have demonstrated that efferocytosis not only involves intricate regulation of “find‐me,” “eat‐me,” and “don't‐eat‐me” signaling molecules but also actively orchestrates inflammation resolution and tissue repair through metabolic reprogramming of amino acids, lipids, and glucose. These findings establish efferocytosis as a central regulator of immune tolerance, suppression of pathological inflammation, and tissue regeneration, offering novel perspectives for understanding disease pathogenesis [4].

The context‐dependent duality of efferocytosis represents a critical area of investigation, with significant implications for both immunology and oncology. Current evidence suggests that effective efferocytosis leads to AC accumulation, triggering autoimmune diseases, cardiovascular disorders, and neurodegenerative conditions [5, 6]. Conversely, tumor cells exploit efferocytosis mechanisms to establish immunosuppressive microenvironments that promote tumor progression [7]. Current research remains fragmented across disease‐specific domains, lacking systematic integration. This necessitates a comprehensive review to synthesize molecular mechanisms, pathological implications, and therapeutic potentials of efferocytosis, thereby establishing theoretical foundations for precision interventions in related diseases.

To provide a comprehensive understanding of efferocytosis, this review is structured under a “mechanism–pathology–disease manifestation–therapy” framework. We begin by detailing the core molecular and metabolic mechanisms that dictate inflammation resolution and tissue repair. Building on this biological foundation, we synthesize current evidence to define four universal hallmarks of efferocytic dysfunction: senescence‐driven impairment, unresolved inflammation, loss of immune tolerance, and defective tissue regeneration. We then examine how these pathological hallmarks drive disease progression across specific cardiovascular, neurological, and oncological contexts. Ultimately, we leverage the dual nature of efferocytosis to propose a context‐dependent directionality therapeutic strategy‐contrasting the need for its enhancement in inflammatory diseases with its targeted inhibition in malignancy. Through this multidisciplinary synthesis, our work provides a systematic reference for advancing both foundational research and the clinical translation of efferocytosis‐targeted precision medicine.

## 2 The Core Machinery and Functional Consequences of Efferocytosis

Efferocytosis is recognized as a critical mechanism for maintaining homeostasis in the human body. Research indicates that it constitutes a continuous and dynamic series of events, primarily involving three sequential steps: recognition, engulfment, and degradation [2, 3]. During each phase, phagocytes undergo significant metabolic reprogramming to supply the energy required for the entire phagocytic process. This mechanism serves not only as an essential component of cellular clearance but also as a fundamental basis for tissue repair and metabolic renewal in humans.

### 2.1 Sequential Machinery of Efferocytosis: From Sensing to Degradation

Efferocytosis initiates with the release of “find‐me” signals by ACs, which serve as molecular beacons to recruit phagocytes. These signals include nucleotides (ATP and UTP), chemokines (CX3CL1), and lipid mediators (sphingosine‐1‐phosphate, S1P), which bind to their respective receptors (P2Y2, CX3CR1, S1PR1) on phagocytes such as macrophages and dendritic cells (DCs) [8, 9, 10]. This chemotactic recruitment is spatially and temporally regulated; for instance, ATP gradients generated via pannexin channels guide phagocytes toward early‐stage ACs, while CX3CL1 sustains migration in later stages [1, 11].

Once proximal to the dying cell, phagocytes transition to the recognition phase, where ACs expose “eat‐me” signals. The most prominent of these signals is phosphatidylserine (PS), a phospholipid normally confined to the inner leaflet of the plasma membrane but actively externalized during apoptosis [12]. Recognition of PS triggers phagocyte engulfment through receptor–ligand interactions. Direct PS binding occurs via phagocyte surface receptors such as T‐cell immunoglobulin and mucin domain‐containing protein 4 (TIM‐4), brain‐specific angiogenesis inhibitor 1 (BAI1), and Stabilin‐2, while indirect recognition involves soluble bridging molecules such as growth arrest‐specific protein 6 (Gas6) and milk fat globule epidermal growth factor 8 (MFG‐E8), which tether PS to TAM family receptors (Tyro3, Axl, MerTK) [13, 14, 15, 16, 17]. Meanwhile, normal cells express “don't‐eat‐me” signals to avoid being mistakenly attacked by the immune system. These signals protect healthy cells from unnecessary attacks by phagocytes such as macrophages through a self‐recognition mechanism.

The ligand–receptor interactions trigger downstream signaling cascades, thereby promoting the digestive process. For instance, BAI1 recruits engulfment and cell mobility (ELMO) dedicator of cytokinesis 180 (Dock180) complexes to activate the Ras‐related C3 botulinum toxin substrate 1 (Rac‐1) signaling pathway, thereby driving cytoskeletal remodeling and forming phagocytic cups that envelop the apoptotic target [18]. Completion of the engulfment process is contingent upon membrane scission, which facilitates the intracellular sequestration of the AC into a phagosome. Subsequent maturation of the phagosome involves fusion with lysosomes, where acid hydrolases degrade apoptotic cargo into nucleotides, lipids, and amino acids, completing the efferocytosis cycle [19].

**FIGURE 1:**
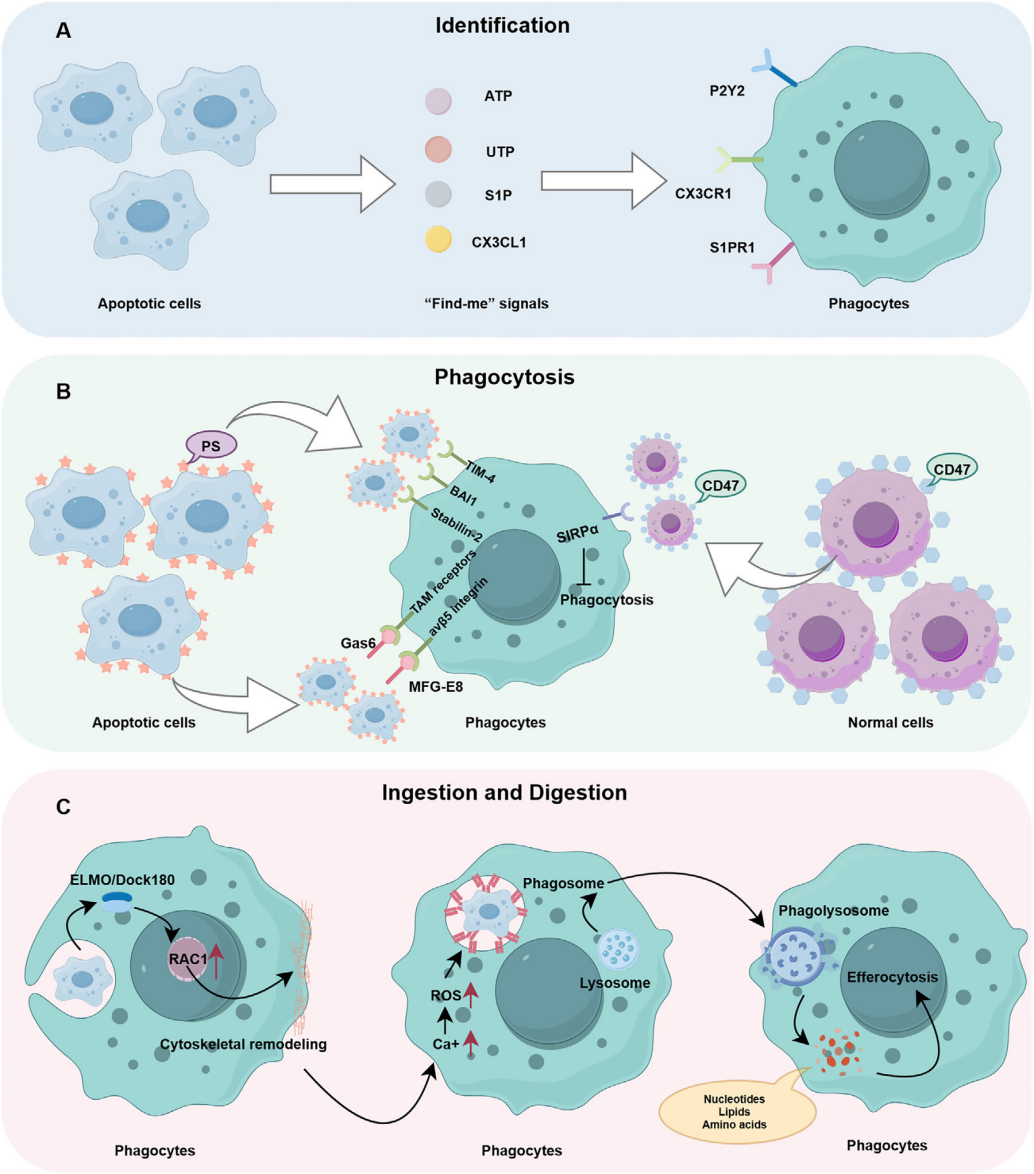
The process of efferocytosis mainly includes three key steps: identification (A), phagocytosis (B), and ingestion and digestion (C). ACs release “find‐me” signals such as ATP, which bind to receptors on the surface of phagocytes and induce their recruitment. Subsequently, phagocytes confirm their targets by recognizing “eat‐me” signals on the surface of apoptotic cells (such as PS), while normal surviving cells express “don't‐eat‐me” signals to avoid being misidentified. After successful recognition, the cell membrane of phagocytes remodels to form a phagocytic cup that encloses the ACs and internalizes them into phagosomes. These phagosomes then fuse with lysosomes to form phagolysosomes, where ACs are degraded into metabolic products. These metabolic products not only participate in cellular metabolism but also act as signaling molecules to drive the next round of efferocytosis. ACs, apoptotic cells; ATP, adenosine triphosphate; BAI1, brain‐specific angiogenesis inhibitor 1; Dock180, dedicator of cytokinesis 180; ELMO, engulfment and cell motility; Gas6, growth arrest‐specific 6; MFG‐E8, milk fat globule‐EGF factor 8; PS, phosphatidylserine; RAC1, Ras‐related C3 botulinum toxin substrate 1; ROS, reactive oxygen species; S1P, sphingosine‐1‐phosphate; SIRPα, signal regulatory protein alpha; TIM‐4, T‐cell immunoglobulin and mucin domain‐containing protein 4; UTP, uridine triphosphate.

In disease contexts, defects in efferocytosis lead to pathological consequences. For example, impaired clearance of ACs in systemic lupus erythematosus (SLE) results in undegraded self‐antigens driving aberrant immune responses, while in atherosclerosis, macrophages’ failure to adequately clear dead foam cells accelerates necrotic core formation and plaque instability [20, 21, 22]. Within the tumor microenvironment (TME), efferocytosis exhibits a dual role: on one hand, tumor‐associated macrophages (TAMs) engulf apoptotic tumor cells to suppress proinflammatory signals, thereby maintaining an immunosuppressive milieu that facilitates tumor immune evasion; on the other hand, specific tumor cells hijack efferocytosis‐related pathways (MerTK receptor activation) to induce macrophages to secrete proangiogenic factors and proproliferative molecules, directly driving tumor growth, metastasis, and therapy resistance [7] (Figure 1).

### 2.2 The Metabolic Reprogramming and Resolution of Inflammation

Efferocytosis plays a dual role in inflammation resolution: it prevents the leakage of proinflammatory intracellular components from secondarily necrotic cells, while actively steering phagocytes toward an anti‐inflammatory and proresolving phenotype through profound metabolic reprogramming. This reprogramming encompasses shifts in lipid, glucose, and amino acid metabolism, which collectively orchestrate the resolution of inflammation [6].

It is well‐established that amino acid metabolism undergoes extensive rewiring in macrophages during efferocytosis, a process intricately linked to inflammation resolution via several complementary pathways [23, 24, 25]. One key mechanism involves the metabolism of AC‐derived arginine by macrophage arginase 1 (Arg1) to ornithine, which ornithine decarboxylase (ODC) further converts to putrescine. Putrescine enhances the HuR‐mediated stabilization of Mcf2 mRNA, leading to upregulation of the Rac1 guanine nucleotide exchange factor Dbl. This promotes Rac1 activation and actin‐dependent internalization of subsequent ACs, thereby optimizing continual clearance and mitigating inflammation‐induced tissue damage [26]. In a second pathway, AC‐derived methionine is converted to S‐adenosylmethionine (SAM) by methionine adenosyltransferase 2A (MAT2A). SAM serves as a methyl donor for DNA methyltransferase 3A (DNMT3A), which methylates the promoter of the dual‐specificity phosphatase Dusp4, thereby suppressing its expression. This alleviates ERK–DUSP4 negative feedback, sustaining ERK1/2 activation, inducing prostaglandin‐endoperoxide synthase 2 (PTGS2/COX2) expression, and promoting prostaglandin E_2_ (PGE_2_)‐dependent secretion of transforming growth factor beta 1 (TGF‐β1), ultimately fostering anti‐inflammatory programming and tissue repair [27]. A third route involves the amino acid sensor GCN2, activated downstream of indoleamine 2,3‐dioxygenase 1 (IDO1)‐mediated tryptophan depletion. Phosphorylation of eukaryotic initiation factor 2α (eIF2α) by GCN2 modulates mRNA translation, specifically inhibiting IL‐12 synthesis by reducing its association with polysomes, while preserving IL‐10 production. This shift promotes a regulatory cytokine profile, suppresses inflammatory T‐cell responses to AC‐derived antigens, and helps maintain immunological tolerance [23]. Moreover, efferocytosis uniquely induces the accumulation of spermidine and spermine in macrophages via Rac1/actin/PI3K‐dependent uptake of extracellular polyamines. These polyamines mediate immunosuppression by mitigating the production of proinflammatory cytokines such as IL‐1β and IL‐6 [28]. Collectively, these amino acid–driven adaptations enhance efferocytic efficiency, promote the secretion of proresolving mediators, and restrain proinflammatory responses, thereby accelerating the resolution of inflammation and promoting tissue homeostasis.

Lipid metabolism also constitutes a crucial axis of metabolic reprogramming during efferocytosis. Upon AC uptake, macrophages internalize membrane‐derived fatty acids, such as long‐chain fatty acids and palmitoylcarnitine, which serve as key metabolic substrates. These fatty acids fuel mitochondrial β‐oxidation (FAO) in a process dependent on carnitine palmitoyltransferases (CPT1/2). FAO supports electron transport chain (ETC) activity, maintaining energy homeostasis for efferocytosis. Concurrently, ETC function elevates intracellular NAD^+^ levels, activating the NAD^+^‐dependent deacetylase SIRT1. SIRT1, in turn, promotes the expression of the anti‐inflammatory cytokine IL‐10 via the transcription factor PBX1, reinforcing an anti‐inflammatory macrophage phenotype and suppressing proinflammatory factors such as tumor necrosis factor‐alpha (TNF‐α) [29]. Beyond energy provision, fatty acid metabolism also generates specialized proresolving lipid mediators (specialized proresolving mediators [SPMs]). The enzyme 12/15‐lipoxygenase (12/15‐LOX) converts AC‐derived polyunsaturated fatty acids (PUFAs), including arachidonic acid (AA) and docosahexaenoic acid (DHA), into SPMs such as lipoxin B_4_ (LXB_4_), resolvin D1 (RvD1), and RvE2 [30, 31]. These SPMs enhance efferocytosis by upregulating phagocytic receptors such as MerTK, and further amplify their own synthesis by promoting the nucleocytoplasmic shuttling of 5‐lipoxygenase (5‐LOX), establishing a positive feedback loop that sustains resolution [32]. SPMs also inhibit neutrophil infiltration, thereby containing inflammatory spread [6, 30]. The MerTK receptor acts as a critical bridge between efferocytosis and lipid mediator production: its signaling inhibits calmodulin‐dependent kinase II (CaMKII), thereby promoting 12/15‐LOX‐mediated SPM generation. Under inflammatory conditions, however, ADAM17‐mediated cleavage of MerTK disrupts this pathway, impairing SPM synthesis and efferocytosis. In contrast, a cleavage‐resistant MerTK variant (MertkCR) maintains SPM production and improves inflammation resolution [33]. Furthermore, FAO‐derived metabolic intermediates can activate the nuclear receptor liver X receptor (LXR), which not only promotes cholesterol efflux via transporters such as ABCA1/ABCG1 but also upregulates efferocytosis‐related molecules like MerTK, thereby preventing chronic inflammation driven by damage‐associated molecular patterns (DAMPs) from uncleared ACs [6, 7, 34]. Thus, lipid metabolic rewiring fine‐tunes inflammation resolution through integrated effects on energy supply, bioactive mediator synthesis, and crosstalk with other regulatory pathways.

Glucose metabolism is similarly redirected toward aerobic glycolysis during efferocytosis. This process triggers a transient glycolytic surge distinct from classical inflammatory glycolysis, driven by Akt‐mediated activation of 6‐phosphofructo‐2‐kinase/fructose‐2,6‐bisphosphatase 2 (PFKFB2) and phosphorylation of thioredoxin‐interacting protein (TXNIP). TXNIP phosphorylation prevents GLUT1 internalization, increasing glucose uptake, while PFKFB2 enhances phosphofructokinase‐1 (PFK‐1) activity via fructose‐2,6‐bisphosphate, collectively boosting glycolytic flux [35]. The resulting lactate functions as a key signaling molecule: it raises cytoplasmic calcium levels, thereby promoting the calcium‐dependent recycling of the efferocytosis receptors MerTK and LRP1 to the cell surface. This receptor replenishment enables sustained AC clearance, curbing secondary necrosis and DAMP release [35]. Efferocytosis also reportedly induces a solute carrier (SLC) transcriptional program centered on SLC2A1 (GLUT1) upregulation, which drives glucose uptake and glycolysis to support actin polymerization, which is required for AC engulfment. Inhibiting SLC2A1 or glycolytic enzymes impairs F‐actin formation and compromises efferocytosis [36]. Extracellular lactate, exported via SLC16A1, further shapes the tissue microenvironment by inducing anti‐inflammatory markers (e.g., TGF‐β1, IL‐10, Mgl1, Mgl2) in neighboring macrophages and suppressing proinflammatory cytokine production [36]. In contrast, the pentose phosphate pathway (PPP) is suppressed during efferocytosis via upregulation of miR‐323‐5p, which targets key PPP enzymes. Pharmacological activation of the PPP or inhibition of miR‐323‐5p impairs AC phagocytosis and exacerbates proinflammatory responses, aggravating lupus‐like pathology in vivo. Building upon this finding, it can be inferred that PPP downregulation is essential for tolerogenic efferocytosis [37]. Glycolytic reprogramming also intersects with other metabolic signals: for instance, glycolytic intermediates synergize with AC‐derived nucleotides to activate mTORC2, driving efferocytosis‐induced macrophage proliferation (EIMP). This expansion of the proresolving macrophage pool enhances AC clearance and anti‐inflammatory cytokine production (e.g., TGF‐β, IL‐10), further accelerating the resolution of inflammation [38].

**FIGURE 2:**
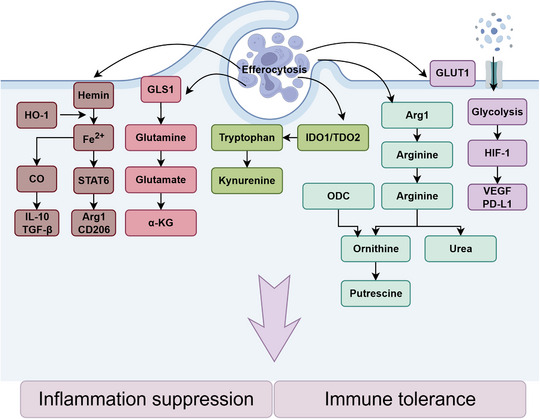
Efferocytosis, a metabolically regulated process, involves phagocytes engulfing ACs and subsequently activating specific metabolic pathways (including glucose metabolism, amino acid metabolism, and iron homeostasis) that facilitate the production of anti‐inflammatory mediators. Notably, Arg1 plays a pivotal role in this immunomodulatory metabolic cascade by orchestrating key biochemical transformations during the resolution phase of inflammation. ACs, apoptotic cells; α‐KG, alpha‐ketoglutarate; Arg1, arginase 1; CD206, cluster of differentiation 206; CO, carbon monoxide; GLS1, glutaminase 1; GLUT1, glucose transporter 1; HIF‐1, hypoxia‐inducible factor 1; HO‐1, heme oxygenase‐1; IDO1, indoleamine 2,3‐dioxygenase 1; IL‐10, interleukin‐10; ODC, ornithine decarboxylase; PD‐L1, programmed death‐ligand 1; STAT6, signal transducer and activator of transcription 6; TDO2, tryptophan 2,3‐dioxygenase 2; TGF‐β, transforming growth factor‐beta; VEGF, vascular endothelial growth factor.

In summary, efferocytosis orchestrates a highly coordinated metabolic transition in macrophages, establishing a biochemical foundation for the resolution of inflammation. A deeper understanding of these metabolic circuits opens promising therapeutic avenues for treating chronic inflammatory diseases (Figure 2).

## 3 The Hallmarks of Defective Efferocytosis in Disease Pathogenesis

In recent years, accumulating evidence has demonstrated that dysregulated efferocytosis is closely linked to the pathogenesis and progression of a wide range of diseases. As a key physiological mechanism for the clearance of ACs, efferocytosis plays a critical role in maintaining tissue homeostasis, suppressing excessive inflammation, and modulating immune responses. Impaired efferocytosis disrupts inflammatory homeostasis, triggers aberrant immune activation, and contributes to tissue damage and disease progression. Overall, dysregulation of efferocytosis exhibits conserved pathophysiological mechanisms across various diseases, including defective clearance of inflammatory cells, autoimmune regulatory imbalance, diminished tissue repair capacity, and secondary necrosis. These pathological alterations may occur independently or concurrently, ultimately leading to disease exacerbation and potentially promoting oncogenesis. Therefore, understanding the mechanisms underlying dysregulation of efferocytosis in pathological contexts has become a major focus of contemporary biomedical research.

### 3.1 Aging Underlies Efferocytosis Failure in Tissue Homeostasis

Aging‐induced impairment of efferocytosis is a complex, multilevel, and multifaceted process. Its core lies in disrupting the delicate balance between prophagocytic “eat‐me” signals and antiphagocytic “don't‐eat‐me” signals, while compromising the intrinsic ability of phagocytes to perform efferocytic functions [39].

First, the most direct negative impact of aging is the downregulation of key receptors on phagocytes that recognize “eat‐me” signals. Tyrosine kinase receptors such as MerTK and Axl are core molecules through which professional phagocytes (including macrophages and microglia) recognize PS exposed on the surface of ACs [40]. During organismal senescence, a significant downregulation of these receptors occurs, effectively compromising the sensory capacity of phagocytes and impeding their ability to detect and clear surrounding ACs. Single‐cell RNA sequencing studies provide more refined evidence for this: in the retinas of aged mice, microglia not only exhibit reduced expression of receptors like MerTK but also show dysfunction of the entire gene network associated with efferocytosis [41]. For instance, the scavenger receptor Msr1 (involved in the internalization of ACs) and the small GTPase Rac2 (which regulates cytoskeletal rearrangement and is crucial for the encapsulation of ACs) both exhibit markedly decreased expression. Critically, senescence‐associated efferocytic failure involves a dual dysregulation: the attenuation of proengulfment ligands is mirrored by the aberrant induction of “don't‐eat‐me” signals, further shielding ACs from clearance [42]. Current evidence suggests that senescent cells, including senescent phagocytes and other senescent cell types, markedly upregulate the surface expression of proteins such as CD47 and CD24 [43, 44]. Interestingly, CD47 binds to the receptor SIRPα (signal regulatory protein alpha) on the phagocyte surface, while CD24 interacts with Siglec‐10, each transmitting potent “don't‐eat‐me” inhibitory signals.

In addition to the aforementioned imbalance at the signal‐recognition level, aging profoundly impairs the intrinsic processing capacity of phagocytes following phagocytosis of ACs, namely, during the degradation phase. Efferocytosis is a complete cycle encompassing recognition, endocytosis, trafficking, and degradation. Age‐related decline in organelle function, particularly mitochondrial dysfunction and impaired autophagic flux, plays a pivotal role in this phase. In aged macrophages, lysosomal degradative capacity is significantly attenuated after phagocytosis of ACs or lipids. This deficit is characterized by insufficient conversion of the autophagy marker LC3 to its active form, LC3‐II, indicating impaired autophagosome formation and fusion with lysosomes [45]. Undegraded AC debris accumulates within phagocytes, and their lipid components form lipid droplets, which in turn exacerbate the metabolic burden and stress status of the cells [45]. This “digestive insufficiency” triggers inflammasome activation, leading to increased secretion of proinflammatory cytokines such as IL‐6 and TNF‐α. Thus, an efferocytotic process that should inherently be anti‐inflammatory and proreparative is transformed into a proinflammatory event in the context of cellular aging.

To summarize, aging impairs efferocytosis through a three‐pronged attack: first, it diminishes the ability of phagocytes to recognize “eat‐me” signals; second, it upregulates the expression of “don't‐eat‐me” signals on senescent cells, inducing a bystander suppression effect; third, it compromises the capacity of phagocytes to degrade apoptotic debris intracellularly, leading to a gain‐of‐function proinflammatory response. The interplay of these mechanisms results in a marked decline in AC clearance, establishing a cellular environment that fosters chronic inflammation and age‐related diseases.

### 3.2 Impaired Inflammation Resolution and Chronic Inflammation

In recent years, researchers have proposed that inflammation is a common pathological basis for many major chronic diseases, including cardiovascular diseases, diabetes, and cancer [46, 47, 48, 49]. A highly conserved and detrimental vicious cycle exists between dysregulated macrophage‐mediated efferocytosis and exacerbated inflammation across diverse chronic inflammatory diseases and tissue injury. This cycle is a central driver of persistent disease progression, with its underlying mechanisms showing notable commonalities across various organ systems and disease models.

The deleterious feedback loop is initiated by a primary defect in efferocytosis, a crucial physiological process for clearing ACs and maintaining tissue homeostasis. Under physiological conditions, ACs release “find‐me” signals such as lysophosphatidylcholine (LPC) and S1P, and expose “eat‐me” signals, including PS on their surface. These signals are recognized by corresponding receptors (G2A, TAM receptor family members Axl/MerTK, CD36, αvβ3 integrin) on macrophages, guiding the noninflammatory recognition, phagocytosis, and degradation of the ACs. Successful efferocytosis not only clears cellular debris but also actively induces macrophages to secrete anti‐inflammatory cytokines such as IL‐10 and TGF‐β. Furthermore, it promotes a phenotypic switch in macrophages from a proinflammatory M1 state toward an anti‐inflammatory/reparative M2 phenotype, thereby actively resolving inflammation [5, 50]. However, under pathological conditions, this precise clearance mechanism is disrupted at multiple levels.

In chronic pancreatitis, the downregulation of the transcription factor Bhlha15 in acinar cells and the consequent reduction in the expression of the phospholipid scramblase ATP8b1 lead to insufficient production of the critical “find‐me” signal LPC, thereby markedly impairing macrophage chemotaxis and clearance capacity [51]. Similarly, within atherosclerotic plaques, elevated expression of CD147 on myeloid cells disrupts TAM receptor‐mediated efferocytic signaling by inhibiting the STAT6 pathway, thereby reducing the production of the key bridging ligand, Gas6 [52]. In the hyperglycemic milieu of diabetic periodontitis, downregulation of SIRT6 in macrophages epigenetically upregulates the miR‐216/217 cluster, which directly suppresses the translation of critical efferocytic effectors such as DEL‐1 and CD36 [53]. Furthermore, the pathological microenvironment can trigger proteolytic events. For instance, in nonalcoholic steatohepatitis (NASH), inflammatory cytokines such as TNF‐α and IL‐1β can activate the ADAM17 protease, leading to ectodomain shedding of the TREM2 receptor on macrophages and consequently impairing their phagocytic function [54]. Analogously, following intracerebral hemorrhage, the inflammatory milieu can compromise the function of the Axl receptor in microglia via a similar mechanism [55]. These diverse molecular events identified across distinct pathologies converge on a common outcome: failure to clear ACs in a timely and efficient manner, leading to their aberrant accumulation within tissues.

Dysfunctional efferocytosis directly triggers the most detrimental step in this vicious cycle: the progression of ACs toward secondary necrosis and the consequent release of abundant DAMPs. In nearly all described pathological contexts, whether in the pancreas, vasculature, liver, heart, or brain, ACs that escape timely clearance eventually lose membrane integrity, leaking endogenous danger signals such as HMGB1, ATP, DNA fragments, and histones [51, 56, 57, 58, 59]. These DAMPs act as potent triggers of inflammatory cascades. They are recognized by pattern recognition receptors (PRRs) on immune cells such as macrophages, including Toll‐like receptors (TLRs) and the intracellular NLRP3 inflammasome [55, 60], leading to activation of canonical inflammatory signaling pathways such as nuclear factor‐kappa B (NF‐κB). This activation, in turn, drives a robust secretion of proinflammatory cytokines, including TNF‐α, interleukin‐1beta (IL‐1β), and interleukin‐6 (IL‐6), culminating in a cytokine storm.

Defective efferocytosis, DAMP release, and inflammation constitute a vicious cycle that, alongside macrophage imbalance, drives the terminal endpoints of fibrosis and organ dysfunction. The persistent inflammatory microenvironment is a powerful driver of fibroblast and stellate cell activation (e.g., pancreatic stellate cells, hepatic stellate cells [HSCs], cardiac fibroblasts). These activated myofibroblasts excessively synthesize and deposit extracellular matrix (ECM) components, primarily collagen, leading to tissue fibrosis, scar formation, and architectural remodeling. A bidirectional promotional relationship exists between inflammation and fibrosis; notably, activated stellate cells can secrete factors such as monocyte chemoattractant protein‐1 (MCP‐1), thereby recruiting additional macrophages, while simultaneously receiving further activation signals from macrophage‐derived factors, such as TGF‐β, thereby establishing another positive feedback loop. Extensive fibrosis progressively replaces functional parenchymal cells, resulting in progressive organ failure, manifested as exocrine and endocrine insufficiency in chronic pancreatitis, heart failure (HF) following cardiac injury, and cirrhosis in NASH. Furthermore, the chronic inflammatory milieu increases genomic instability, significantly elevating the risk of carcinogenesis.

### 3.3 Loss of Immunological Tolerance and Autoimmunity

Under physiological conditions, efferocytosis serves as a crucial mechanism for maintaining immune tolerance. By efficiently clearing ACs generated daily, efferocytosis not only prevents the leakage of intracellular self‐antigens (such as nucleoproteins and DNA) but also actively induces an anti‐inflammatory microenvironment: upon recognizing the “eat‐me” signals on the surface of ACs, phagocytes (including macrophages and DCs) secrete anti‐inflammatory factors (such as TGF‐β and IL‐10), and promote the differentiation of regulatory T cells (Tregs) [61]. This process inhibits autoreactive immune responses and maintains peripheral tolerance. However, under pathological conditions, dysregulated efferocytosis disrupts this delicate balance and becomes a core driver in the initiation and progression of autoimmune diseases. Accumulating evidence indicates that defective efferocytosis promotes the accumulation of ACs, exposure of self‐antigens, activation of chronic inflammation, and breakdown of immune tolerance through multiple mechanisms, ultimately triggering autoimmune dysregulation [62].

It is now understood that the dysregulation of immune tolerance in various autoimmune disorders is fundamentally driven by deficient efferocytosis, which results in the widespread exposure of self‐antigens. For instance, in SLE, macrophages from SLE patients exhibit a marked reduction in their capacity to clear ACs, driven by multiple factors, including genetic defects in the complement component C1q (C1q‐knockout mice develop an SLE‐like phenotype), downregulated expression of MFG‐E8, or the production of autoantibodies against SCARF1 [21, 63, 64]. Unclear ACs accumulate in lymphoid tissues, releasing self‐antigens such as nucleosomes and double‐stranded DNA (dsDNA). These antigens are captured by follicular DCs and presented to B cells, driving the production of autoantibodies (such as anti‐dsDNA antibodies) [62, 65]. The resulting immune complexes deposit in organs such as the kidneys, exacerbating tissue damage. In arthritis, efferocytosis defects primarily impact the joint microenvironment: impaired function of MerTK or Axl in synovial macrophages leads to insufficient clearance of apoptotic neutrophils [66, 67]. These uncleared cells release proteases and proinflammatory cytokines, which promote cartilage destruction and bone erosion. Besides, defective efferocytosis amplifies interferon (IFN) signaling via activation of the STING‐cGAS pathway, recruiting autoreactive T cells and thereby disrupting immune homeostasis.

In contrast to other diseases, within the TME, efferocytosis, a physiological process originally intended to maintain tissue homeostasis, is “hijacked” by tumor cells [68]. Its function undergoes a fundamental reversal, shifting from inducing immune tolerance to establishing and sustaining a profound state of immune suppression, thereby emerging as a key driver of tumor immune escape. The core of this process is that when phagocytes, such as TAMs, clear apoptotic tumor cells, they do not elicit antitumor immunity. Instead, they trigger a series of intracellular reprogramming events that actively suppress the immune system. Efferocytosis directly induces the secretion of immunosuppressive factors. Through signaling pathways mediated by TAM family receptors such as MerTK and Axl, macrophages that have phagocytosed ACs are polarized into an M2‐like phenotype [69]. They secrete large amounts of key anti‐inflammatory factors, including TGF‐β and IL‐10, which directly inhibit the activation and function of cytotoxic CD8^+^ T cells and promote the generation of Tregs [7]. This creates a potent local immunosuppressive microenvironment.

These findings underscore the necessity of investigating efferocytosis within disease‐specific contexts, as its immunomodulatory impact is likely divergent and governed by unique underlying mechanisms.

### 3.4 Defective Tissue Repair and Fibrosis

In various chronic disease models, the dysregulation of efferocytosis has been identified as a core driver of abnormal tissue repair and the progression of pathological fibrosis [70, 71]. Its pathophysiological process typically begins with a “vicious cycle”: tissue damage triggers the generation of numerous ACs. Due to impaired efferocytosis, these ACs cannot be cleared in a timely manner, leading to secondary necrosis and the release of DAMPs [72]. These DAMPs continuously stimulate immune cells, such as macrophages, by activating PRRs (TLRs) and NLRP3 inflammasomes. This stimulation results in excessive production of proinflammatory cytokines, including IL‐1β, TNF‐α, and IL‐6, thereby maintaining a state of chronic low‐grade inflammation [73]. This persistent inflammatory microenvironment not only directly impairs the repair capacity of tissue‐regenerating cells but also potently drives fibroblast and stellate cell activation. Therefore, through establishing the vicious cycle of “ACs accumulation–DAMP release–chronic inflammation‐sustained tissue damage,” efferocytosis dysfunction creates the necessary conditions for the development of fibrosis [74].

The core mechanism by which efferocytosis dysfunction drives fibrosis lies in its disruption of macrophages’ normal immunoregulatory and proreparative functions, transforming macrophages into a profibrotic phenotype. Under physiological conditions, successful efferocytosis induces macrophages to secrete anti‐inflammatory factors (such as IL‐10 and TGF‐β) and proresolving mediators. It also promotes the polarization of macrophages toward the reparative M2 phenotype, thereby coordinating the resolution of inflammation and tissue remodeling. However, in pathological states, this sophisticated feedback regulation is disrupted. First, sustained efferocytic burden or specific types of ACs (e.g., apoptotic type II alveolar epithelial cells) directly induce macrophages to highly express profibrotic factors, including the active form of TGF‐β1 and Arg‐1 [70, 75, 76]. TGF‐β1 is one of the most potent factors that activate fibroblasts and promote excessive deposition of the ECM. Second, abnormal metabolic reprogramming, an essential process for efferocytosis, further exacerbates fibrosis. For instance, deficiency of the Atp6v0d2 gene in hepatic macrophages triggers endoplasmic reticulum stress and disorders in cholesterol metabolism. This impairs their efferocytic capacity, leads to the accumulation of apoptotic hepatocytes, and thereby activates HSCs to drive liver fibrosis [77]. Similarly, in pulmonary fibrosis, MERTK expression is abnormally elevated. Although this elevation attempts to enhance efferocytosis, its downstream signaling paradoxically promotes TGF‐β production and fibroblast activation. Meanwhile, mitochondrial dysfunction impairs negative feedback mechanisms, allowing profibrotic signals to remain dominant [73]. Furthermore, emerging studies have revealed the role of mechanosensing in this process: tissue stiffening enhances efferocytosis by activating the Piezo1 channel on macrophages, thereby promoting fibrosis resolution. Conversely, the failure of this mechanosensing–efferocytosis axis accelerates fibrosis progression [78]. These pieces of evidence collectively indicate that through multiple mechanisms, including metabolic disorders, imbalanced phenotypic transition, and defective signal feedback, efferocytosis dysfunction transforms macrophages from coordinators of repair into drivers of fibrosis.

Finally, the combined effect of the aforementioned cellular and molecular events leads to pathological fibrosis, characterized by excessive ECM deposition and tissue scarring. This fibrotic repair replaces normal tissue regeneration and gradually impairs organ function, such as impaired pulmonary gas exchange in idiopathic pulmonary fibrosis (IPF), liver cirrhosis and liver failure in metabolic dysfunction‐associated steatohepatitis (MASH), and cardiac remodeling and HF following myocardial infarction (MI) [59].

**FIGURE 3:**
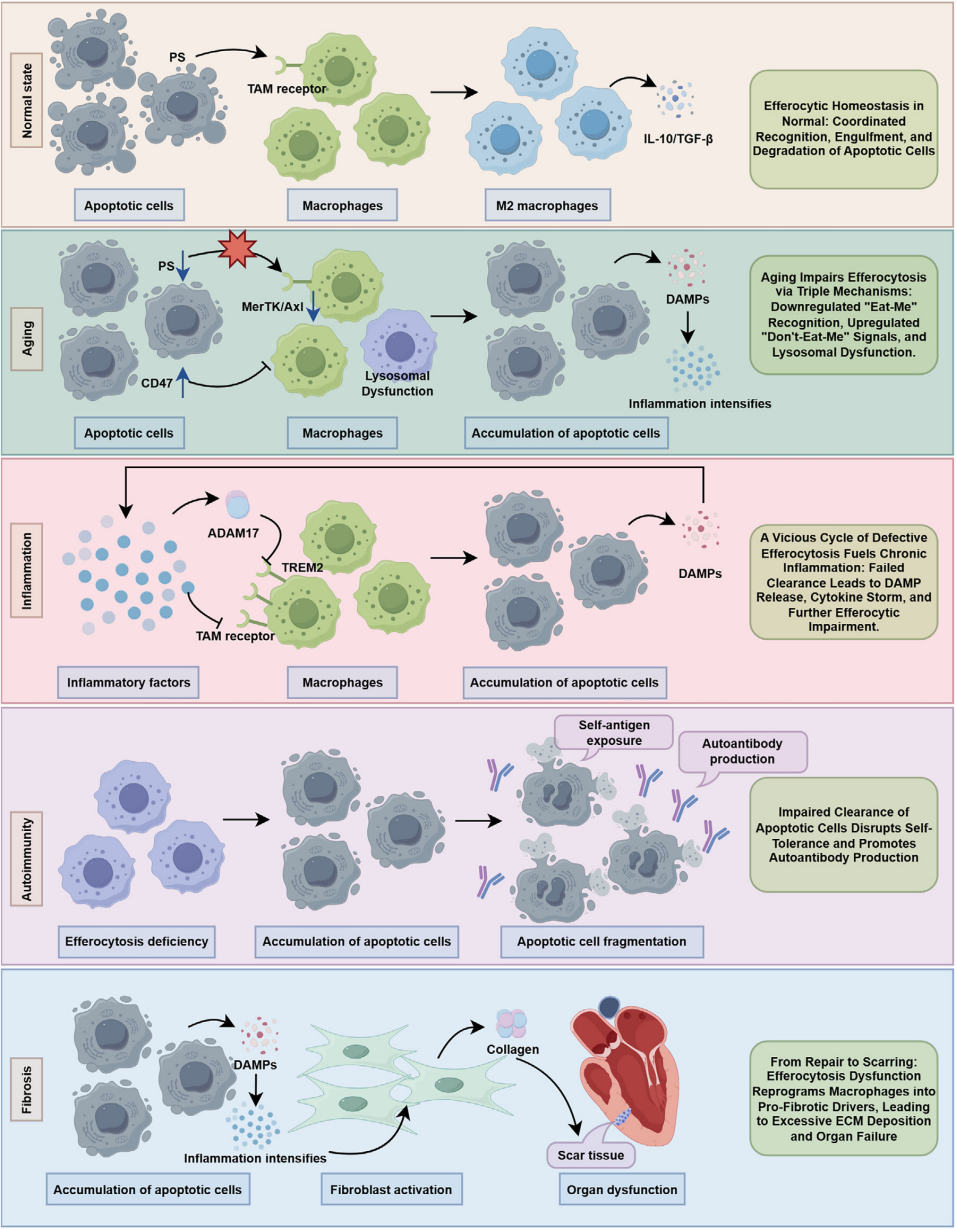
Efferocytosis, a critical homeostatic mechanism for metabolic homeostasis, ensures the clearance of ACs to maintain tissue integrity. However, dysregulated efferocytosis contributes to multiple pathological cascades, including: (1) senescence‐driven suppression of efferocytic capacity, (2) the inflammatory–efferocytosis–inflammation amplification feedback loop, (3) autoimmune dysregulation due to defective ACs clearance, and (4) impaired tissue repair with pathological fibrosis. These interconnected pathophysiological disruptions collectively constitute the pathological basis for the progression of numerous diseases. ACs, apoptotic cells; ADAM17, A disintegrin and metalloproteinase 17; DAMPs, damage‐associated molecular patterns; ECM, extracellular matrix; IL‐10, interleukin‐10; PS, phosphatidylserine; TGF‐β, transforming growth factor‐beta; TREM2, triggering receptor expressed on myeloid cells 2.

In summary, dysregulation of efferocytosis represents a core pathological mechanism underlying the initiation and progression of multiple diseases. This chapter systematically elaborates that impaired efferocytic function drives disease pathogenesis through four key pathological hallmarks: 1. Age‐related clearance impairment (imbalanced phagocytic recognition signals and reduced degradative capacity lead to AC accumulation and chronic inflammation); 2. Impaired inflammation resolution (defective clearance triggers the release of DAMPs and amplification of inflammatory cascades, forming a vicious cycle of efferocytosis deficiency–exacerbated inflammation); 3. Loss of immune tolerance (exposure of self‐antigens induces autoimmune responses, or efferocytosis is co‐opted as an immunosuppressive mechanism within the TME); 4. Defective tissue repair and fibrosis (a persistent inflammatory microenvironment activates fibroblasts, promoting excessive ECM deposition and organ fibrosis). Collectively, these alterations undermine organ integrity and elevate malignancy risk. Delineating the multilayered drivers of impaired efferocytosis identifies transformative therapeutic opportunities for managing inflammatory and fibrotic pathologies (Figure 3).

## 4 Dysregulated Efferocytosis in Specific Disease Contexts

**FIGURE 4:**
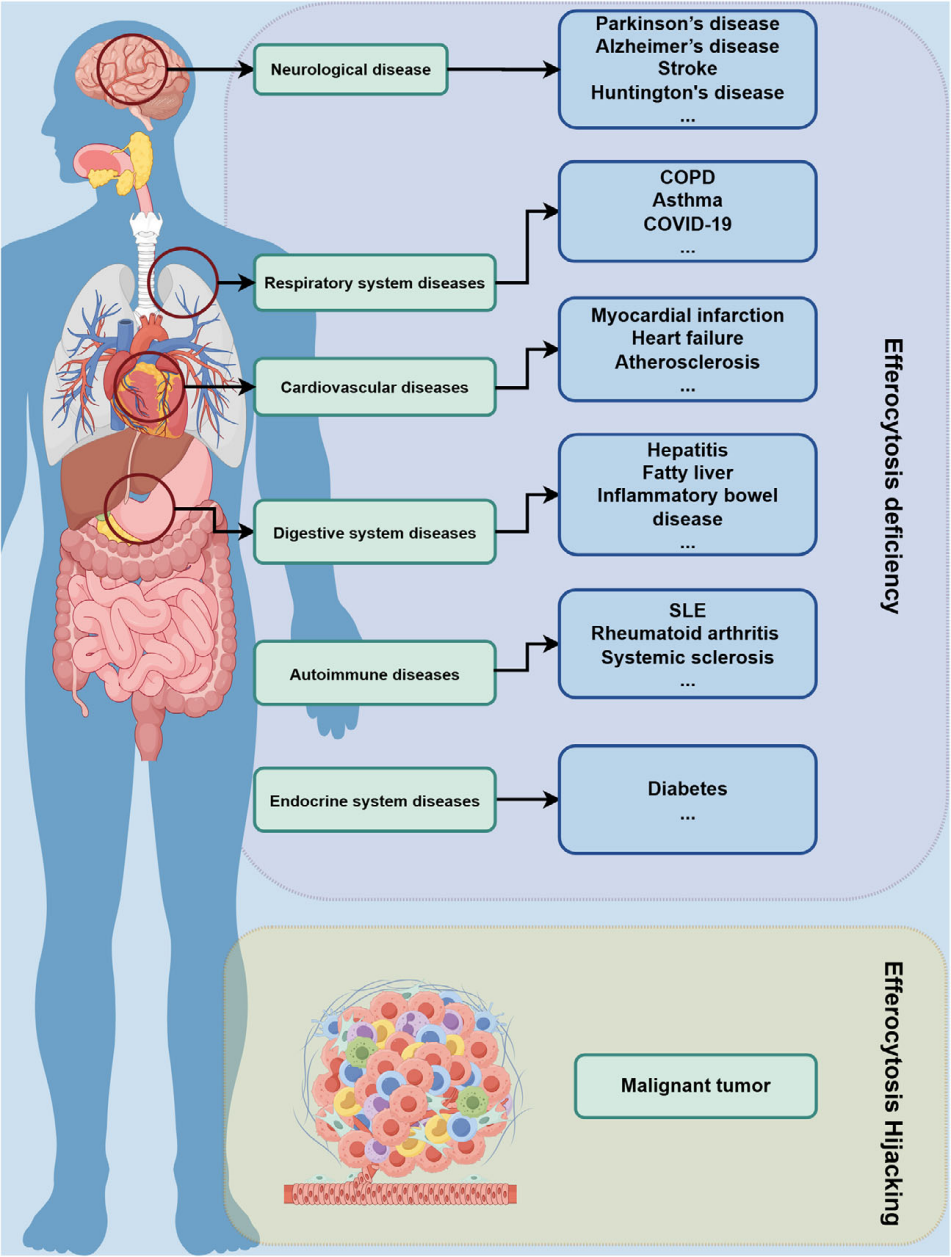
Dysregulation of efferocytosis constitutes a pathophysiological nexus linking diverse human diseases. In chronic inflammatory and autoimmune disorders, defective efferocytic clearance exacerbates disease progression through persistent accumulation of ACs and sustained proinflammatory signaling. Conversely, in malignancies, tumor cells actively exploit the efferocytic machinery to establish an immunosuppressive niche, thereby subverting this homeostatic process to facilitate oncogenic progression. COPD, chronic obstructive pulmonary disease; SLE, systemic lupus erythematosus.

Based on the aforementioned discussion, impaired efferocytosis can lead to a spectrum of aberrant pathophysiological phenomena, including inflammatory disorders, immune dysregulation, and fibrosis. These interconnected pathological processes are fundamental to the initiation or progression of various diseases. A comprehensive dissection of context‐specific efferocytic failure and its pathological consequences significantly advances our understanding of disease pathogenesis. Furthermore, it establishes atheoretical framework and identifies precise molecular targets for the development of efferocytosis‐modulating therapies [79] (Figure 4).

### 4.1 Cardiovascular Disease

#### 4.1.1 Atherosclerosis

In cardiovascular diseases, especially in atherosclerosis, researchers have reported numerous abnormalities related to efferocytosis, which contribute to the development and progression of the disease. In healthy arteries, macrophages efficiently clear ACs, preventing leakage of their contents and subsequent inflammation, thereby maintaining plaque stability. However, this balance is disrupted in the chronic inflammatory environment characteristic of atherosclerosis. Excessive lipid accumulation within plaques, oxidative stress, and the massive release of inflammatory factors not only cause cells to die in more severe ways (such as necroptosis and pyroptosis) but also directly impair the efferocytic capacity of macrophages [80, 81, 82].

As mentioned earlier, ACs expose PS, an evolutionarily conserved “eat‐me” signal. However, many cells undergoing lytic cell death (such as necroptosis and pyroptosis) are abundant within atherosclerotic plaques, and their plasma membrane integrity is compromised. Although these cells also expose PS, the accessibility of this signal is due to increased membrane permeability rather than to active phospholipid flipping, which may compromise the specificity and efficiency of subsequent recognition processes [83, 84]. In addition, studies have demonstrated that the expression of CD47, a well‐characterized “don't‐eat‐me” signal, is markedly upregulated in atherosclerotic plaques of both humans and mice, which directly abrogates the progression of efferocytosis [85, 86, 87]. Meanwhile, accumulation of proinflammatory cytokines such as TNF‐α can further upregulate CD47 expression via the NF‐κB signaling pathway, thereby exacerbating impairment of AC clearance.

Meanwhile, researchers have found that phagocytes, primarily macrophages, exhibit marked functional impairment in atherosclerosis. Studies have confirmed that CD147, derived from myeloid cells (mainly macrophages), is highly expressed in atherosclerotic plaques and is a crucial pathogenic factor in atherosclerosis. CD147 activates the TRAF6‐IKK‐IRF5 signaling pathway, thereby driving macrophage polarization toward the proinflammatory M1 phenotype and upregulating inducible nitric oxide synthase (iNOS) expression. This subsequently promotes the production of nitric oxide and reactive nitrogen species (RNS), exacerbating inflammatory responses. In the inflammatory microenvironment, CD147 inhibits the expression and secretion of Gas6. As a key bridging molecule mediating the clearance of ACs by macrophages, Gas6 downregulation directly induces efferocytic dysfunction, which impairs the efficient clearance of ACs and leads to their accumulation, forming a necrotic core, thereby accelerating disease progression [52]. Another study consistently identified macrophage PKM2 as a pivotal pathogenic factor in atherosclerosis, where it drives disease progression by enhancing inflammatory responses and inhibiting LRP‐1‐mediated efferocytosis [88]. Another study reported that complement factor H (CFH) worsens atherosclerosis by restricting intracellular C3 consumption in macrophages, thereby suppressing LAP‐mediated efferocytosis. This impairs clearance of ACs, expands the necrotic core, and perpetuates inflammation, accelerating disease progression [89].

#### 4.1.2 Myocardial Infarction

MI represents a life‐threatening cardiovascular disorder characterized by a cascade of core pathological events: myocardial ischemia, massive cardiomyocyte death, and subsequent robust inflammatory responses. In this pathological process, macrophage‐mediated efferocytosis serves as a pivotal mechanism that clears apoptotic cardiomyocytes, resolves inflammation, and initiates tissue repair programs. Impaired efferocytosis directly leads to the accumulation of uncleared ACs, which in turn causes a failure of timely inflammatory resolution. This pathological cascade exacerbates myocardial injury, promotes adverse ventricular remodeling, and ultimately impairs cardiac functional recovery, potentially progressing to HF [90, 91].

Accumulating evidence demonstrates that macrophages play a pivotal role in the repair process following MI via efferocytosis. On the one hand, this process relies on the scavenger receptor CD36 and the STAT6 signaling pathway, which trigger macrophages to express and secrete VEGFC [92]. Consequently, this promotes cardiac lymphangiogenesis and improves cardiac function. On the other hand, TREM2^+^ macrophages can also sense ACs via efferocytosis, thereby activating the TREM2‐SYK‐SMAD4 pathway and inhibiting SLC25A53 transcription. Downregulation of SLC25A53 reduces mitochondrial NAD^+^ transport, leading to dysfunction of the tricarboxylic acid (TCA) cycle. This metabolic reprogramming further promotes the accumulation of the immunometabolite itaconate, which contributes to cardiac repair by suppressing cardiomyocyte apoptosis and promoting fibroblast proliferation [93]. Besides, under the pathological context of MI, macrophages may exhibit mitochondrial dysfunction—for instance, deficiency of Ndufs4, a key protein of mitochondrial complex I. This deficit impedes the transition of macrophages toward the reparative phenotype, thereby exacerbating inflammation, inhibiting tissue repair, and ultimately resulting in increased risks of cardiac rupture, progressive cardiac dysfunction, and elevated mortality [94]. Therefore, targeting macrophage‐mediated efferocytosis to restore its physiological function holds considerable promise as a key therapeutic strategy for improving post‐MI outcomes.

### 4.2 Autoimmune Disease

#### 4.2.1 Systemic Lupus Erythematosus

SLE is a chronic autoimmune disease characterized by the loss of immune tolerance, autoantibody production, and multiorgan damage, among which lupus nephritis (LN) represents a prevalent and severe complication. Recent studies have demonstrated that the efferocytosis defect of macrophages plays a central role in the pathogenesis of SLE [95, 96, 97].

In the SLE microenvironment, the pivotal pathological cascade is triggered by the massive release of endogenous DAMPs, such as HMGB1 and cell‐free nucleic acids, resulting from tissue injury or aberrant cell death [21, 98]. These endogenous “danger signals” can be recognized and bound by TLRs, particularly TLR7 and TLR9, which are expressed on the surface of innate immune cells, including macrophages. Aberrant overexpression and activation of TLRs are hallmark features of lupus pathogenesis. Subsequent TLR signaling cascades markedly upregulate TonEBP expression, a transcription factor in macrophages. Once activated, TonEBP exerts its transcriptional regulatory function, directly suppressing the expression of a repertoire of key genes essential for efferocytosis, including the bridging molecule Gas6 and the scavenger receptor CD36. As an elaborate physiological process responsible for the timely clearance of ACs, efferocytosis is impaired by the suppression of the expression of these critical molecules, which directly compromises the phagocytic capacity of macrophages for ACs and thus culminates in efferocytosis deficiency. Ultimately, the generated autoantibodies bind to self‐antigens to form immune complexes, which deposit in organs such as the kidneys, recruit additional inflammatory cells, and induce severe end‐organ damage, including LN [99].

Separately, another study has identified an SLE‐associated genetic variant, NCF1‐R90H, which impairs the function of the NADPH oxidase complex and reduces the production of ROS. In macrophages, the NCF1‐H90 variant compromises phagosome acidification (a process dependent on the Hv1 proton channel) and proteolytic function, leading to the accumulation of AC remnants and subsequent efferocytosis deficiency. This defect further upregulates CD40 expression on the macrophage surface, thereby driving the differentiation and proliferation of T follicular helper 2 (Tfh2) cells while inhibiting T follicular regulatory (Tfr) and Tfh1 cells. These disruptions ultimately result in B‐cell hyperactivation, autoantibody production, and immune complex deposition in the kidneys [100]. Overall, efferocytosis deficiency represents one of the core pathogenic mechanisms underlying SLE. It disrupts the body's immune tolerance to self‐antigens, initiates autoimmune responses, and drives disease progression through sustained antigen exposure and inflammatory signaling.

#### 4.2.2 Other Autoimmune Diseases

Beyond SLE, dysregulation of efferocytosis is also closely implicated in the pathogenic mechanisms of various other autoimmune diseases. Using Sjögren's syndrome (SS) as a paradigm, efferocytosis deficiency plays a pivotal role in its pathogenesis and shares partial immunopathological pathways with SLE. Studies have demonstrated that monocyte‐derived macrophages from SS patients exhibit a marked impairment in their capacity to clear ACs, and this defect is inversely correlated with disease activity. The primary underlying mechanism lies in the presence of inhibitory IgG antibodies in patients, which can interfere with the efficient clearance of ACs via opsonization [101]. Notably, SS‐specific autoantibodies (anti‐SSA/Ro and anti‐SSB/La) can bind to apoptotic cardiomyocytes, thereby impeding their effective phagocytic clearance. This mechanism directly elucidates the etiology of congenital heart block observed in neonates born to SS mothers [102]. The critical importance of efferocytosis has been further validated in animal models: Mer tyrosine kinase receptor‐deficient mice manifest SS‐like phenotypes, including lymphocytic infiltration of salivary glands and autoantibody production [103]. In addition, macrophages from SS‐susceptible mice display an aberrant proinflammatory response subsequent to AC engulfment. Transcriptomic analyses have revealed that genes associated with pathways such as the type I IFN signaling cascade are significantly upregulated, thereby exacerbating local inflammatory reactions [104].

In rheumatoid arthritis (RA), efferocytosis is regarded as a potential disease‐alleviating mechanism. Studies have revealed the presence of a specialized subset of efferocytic macrophages (MERTK^+^CD206^+^ in humans) lining the joint synovium. These cells predominate in healthy individuals but show a marked reduction in proportion in patients with active RA, and this reduction is inversely correlated with DAS28. Upon entering disease remission, the proportion of this cell subset rebounds, suggesting that these macrophages may promote the resolution of inflammation by clearing ACs and secreting the anti‐inflammatory cytokine IL‐10 [105]. Animal experiments have demonstrated that the Mer and Axl receptors, along with their ligands Gas6 and Protein S, confer joint protection, while the deficiency of these molecules exacerbates arthritic pathology [106, 107]. In‐depth elucidation of the specific mechanisms by which efferocytosis deficiency drives immune dysregulation will provide a crucial theoretical basis for the development of targeted therapeutics against autoimmune diseases.

### 4.3 Neurological Disease

#### 4.3.1 Neurodegenerative Diseases

Inflammation has long been recognized as a primary driver of neurodegenerative diseases, a concept that has received substantial support from recent advances in the field [108, 109, 110]. Neuroinflammation and immunity have been identified as a shared pathophysiological basis for multiple central nervous system (CNS) disorders, including Alzheimer's disease (AD) and Parkinson's disease (PD). The core mechanism underlying this association lies in the aberrant activation of brain‐resident immune cells (microglia), which triggers chronic, destructive inflammatory responses and ultimately accelerates neuronal degeneration. Further investigations have revealed that impaired efferocytosis within the nervous system is a crucial contributor to persistent inflammation. A growing body of experimental evidence indicates that defective efferocytic function hinders the timely clearance of ACs, thereby amplifying inflammatory signaling cascades [111, 112]. This mechanism underscores the pivotal role of the “efferocytosis impairment–inflammation exacerbation–disease progression” pathological axis in the initiation and progression of neurodegenerative diseases.

In AD, impaired efferocytosis is recognized as a core mechanism driving disease progression. The pathological hallmarks of AD include amyloid‐β (Aβ) plaque accumulation, neurofibrillary tangles, and chronic neuroinflammation, with microglial dysfunction directly contributing to reduced efferocytic efficiency [113]. Studies have demonstrated that in the AD milieu, sustained exposure of microglia to pathological stimuli such as Aβ induces a state of chronic activation, accompanied by decreased expression or impaired function of their cognate receptors (TREM2 and MerTK) [114, 115, 116, 117]. This compromise impairs the efficient clearance of ACs, leading to the accumulation of cellular debris, secondary necrosis, and the release of proinflammatory cytokines (TNF‐α and IL‐1β). Consequently, neuroinflammation is exacerbated, thereby forming a deleterious vicious cycle. Furthermore, impaired efferocytosis compromises the clearance of Aβ plaques: under physiological conditions, microglia are capable of phagocytosing Aβ aggregates; however, in the context of AD, the phagocytic capacity of microglia is attenuated, and subsequent plaque accumulation further triggers inflammatory responses, thereby accelerating neuronal damage [117, 118]. During the final phagolysosomal digestion phase of efferocytosis, impaired fusion between phagosomes and lysosomes leads to the accumulation of undegraded cellular debris. DAMPs released from this debris activate proinflammatory signaling pathways, including NF‐κB. For instance, aberrant activation of NF‐κB upregulates the expression of proinflammatory cytokines while concomitantly suppressing the expression of receptors such as MerTK, thereby further impairing efferocytic capacity and consolidating the aforementioned vicious cycle [119, 120, 121].

Similarly, during the pathological progression of PD, microglial dysfunction represents a pivotal driver of disease advancement. Such functional impairment is primarily characterized by reduced clearance capacity of pathological proteins and uncontrolled amplification of neuroinflammatory responses. When aberrantly aggregated α‐synuclein (α‐syn), the core pathological hallmark of PD, is released into the extracellular space, it can be recognized by microglia as a DAMP [122]. Studies have demonstrated that pathological α‐syn fibrils directly bind to the receptor for advanced glycation end products (RAGE) on the microglial surface, thereby potently triggering cellular inflammatory responses [123]. Concurrently, the binding of α‐syn to the FcγRIIB receptor suppresses the phagocytic function of microglia, impairing their ability to eliminate pathological proteins [124]. Furthermore, α‐syn can activate the NLRP3 inflammasome via the Fyn kinase and CD36 receptor pathway, leading to the massive release of potent proinflammatory cytokines such as IL‐1β [125]. Collectively, these processes form a vicious cycle: the failure of α‐syn clearance mechanisms, coupled with the persistent exacerbation of neuroinflammation, further accelerates the degeneration of dopaminergic neurons and the progression of PD.

#### 4.3.2 Stroke

Following an ischemic stroke, efferocytosis mediated by infiltrating peripheral macrophages and brain‐resident microglia is critical for clearing dying neurons, restricting inflammatory spread, and maintaining homeostasis in the CNS [126, 127]. Timely and efficient efferocytosis can limit secondary injury, facilitate inflammatory resolution and tissue repair, thereby improving neurological functional outcomes. Conversely, impaired efferocytic function leads to the accumulation of cellular debris from dead cells, which in turn triggers persistent inflammatory responses, exacerbates brain tissue damage, and ultimately results in poor neurological recovery.

When efferocytosis is compromised, uncleared ACs undergo secondary necrosis, releasing a large array of DAMPs. These DAMPs can sustain the activation of PRRs, triggering robust proinflammatory signaling cascades and causing uncontrolled sterile inflammation, which ultimately contributes to infarct expansion [128]. Studies have demonstrated that CD300A (an inhibitory receptor) and CD300B (an activating receptor) competitively bind PS during efferocytosis. CD300A recruits phosphatases such as SHP‐1 via its immunoreceptor tyrosine‐based inhibitory motif (ITIM) domain, thereby suppressing efferocytic activity mediated by the CD300B–DAP12–Syk signaling axis. In the hyperacute phase of ischemic stroke, excessive activation of CD300A inhibits efferocytosis; in contrast, blockade of CD300A enhances the phagocytic capacity of monocytes/macrophages toward dead cells, which is conducive to improved prognosis [129]. Furthermore, as a key transcription factor, STAT6 exerts a central role in promoting the polarization of macrophages/microglia toward the M2 anti‐inflammatory phenotype and enhancing efferocytosis after ischemic stroke. STAT6 facilitates efferocytosis by upregulating the expression of downstream target genes, such as Arg1. Studies have confirmed that STAT6‐knockout mice exhibit more severe brain tissue damage, reduced capacity for dead cell clearance, and exacerbated neurological deficits following ischemic stroke, which further underscores the critical role of STAT6 in the regulation of efferocytosis [130].

## 5 The Dual Role of Efferocytosis in Tumor

As mentioned above, efferocytosis, the core physiological process for clearing ACs, is subtly hijacked by tumor cells in the TME. Its role has shifted from maintaining homeostasis to becoming a key factor driving malignant progression [1, 131]. The core of this transformation lies in the immunosuppression and metabolic remodeling triggered by efferocytosis. Growing clinical evidence indicates that efferocytosis‐related genetic signatures are significantly associated with poor prognosis in patients with various cancers [7, 132, 133, 134, 135]. High‐risk patients are usually accompanied by the infiltration of M2‐type TAMs and elevated tumor mutation burden, which directly confirms their protumorigenic function.

The mechanisms by which efferocytosis promotes tumor progression are multilayered. First, it directly reshapes the immunosuppressive microenvironment. TAM are polarized into the immunosuppressive M2 phenotype through efferocytosis mediated by receptors such as MerTK and Axl [136, 137]. This process activates nuclear receptor signaling pathways, including peroxisome proliferator‐activated receptor gamma (PPARγ) and LXR, which, in turn, induce macrophages to secrete large amounts of immunosuppressive factors such as IL‐10 and TGF‐β [138]. These factors directly inhibit the function of cytotoxic CD8^+^ T cells, creating conditions for tumor immune evasion [139]. Studies have confirmed that during liver metastasis of pancreatic cancer, macrophages phagocytose apoptotic tumor cells through efferocytosis and upregulate Arg1 expression via the CFTR/LXRα/RXRα axis [140, 141]. This rapidly depletes arginine in the microenvironment, thereby disrupting the metabolism and function of CD8^+^ T cells, establishing an “immune‐privileged” niche in the liver and promoting the colonization of metastatic lesions [141]. A similar vicious cycle has also been observed in malignant pleural effusion models: Axl/MerTK‐dependent efferocytosis induces macrophages to secrete IL‐10, which further promotes DC expression of TIMP1. Inhibition of matrix metalloproteinase activity drives vascular remodeling and fluid accumulation, ultimately accelerating tumor dissemination [142]. In addition, efferocytosis can directly stimulate tumor proliferation. For example, it activates the NLRP3 inflammasome signaling pathway to promote the secretion of IL‐1β, thereby directly driving tumor cell growth [143].

In addition to macrophages, neutrophils and DCs are important executors of efferocytosis, but their functions are distorted by tumors in the TME. After phagocytosing ACs, neutrophils polarize into a tumor‐promoting N2 phenotype [144, 145]. They secrete proangiogenic factors such as vascular endothelial growth factor (VEGF) and hepatocyte growth factor (HGF), as well as ECM remodeling enzymes like matrix metalloproteinase 9 (MMP9), which directly promote tumor angiogenesis, invasion, and metastasis. More critically, activated neutrophils highly express Arg1 to consume arginine, an essential nutrient for T‐cell function, and secrete TGF‐β, IL‐10, and PGE_2_, thereby directly inhibiting the activation and proliferation of T cells [146]. In the bloodstream, neutrophils can also form clusters with circulating tumor cells (CTCs). By releasing neutrophil extracellular traps (NETs) and prosurvival factors, they protect CTCs, helping them resist blood flow shear stress and promote distant metastasis [147].

The efferocytic behavior of DCs exhibits context‐dependent dual functions. In the context of acute infection, DCs can effectively perform antigen cross‐presentation by efferocytosing ACs, thereby activating CD8+ T cells. For example, annexin A1 has been shown to enhance the efferocytic efficiency and antigen‐presenting ability of DCs, thus promoting antipathogen immunity [148]. However, in the chronic inflammatory environment of tumors, continuous efferocytic signals drive DCs to transform into a tolerogenic phenotype. The underlying mechanisms include inhibiting the TLR/NF‐κB signaling pathway, reducing the production of proinflammatory factors such as IL‐12, increasing IL‐10 secretion, and inducing the differentiation of regulatory T cells, thereby establishing and maintaining immune tolerance [149, 150, 151]. This functional switch is a crucial link in tumor immune escape.

**FIGURE 5:**
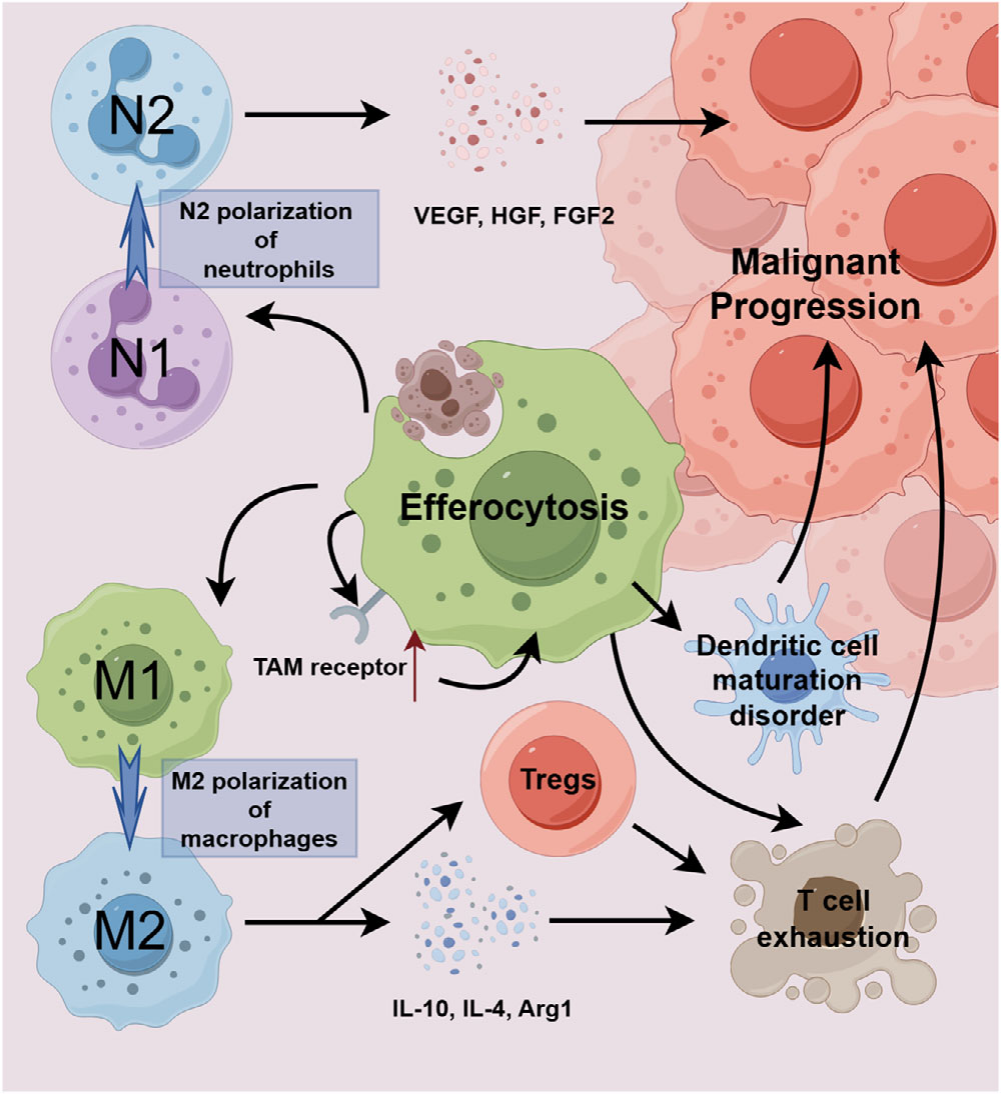
The process of efferocytosis can promote the expression of various immunosuppressive factors (such as TGF‐β) by activating the PPARγ/LXR signaling pathway. These immunosuppressive factors can induce the polarization of macrophages to the M2‐type and neutrophils to the N2 type, further intensifying the formation of an immunosuppressive microenvironment, interfering with T‐cell function, and ultimately facilitating tumor immune escape. Arg1, arginase 1; FGF2, fibroblast growth factor 2; HGF, hepatocyte growth factor; IL‐10, interleukin‐10; IL‐4, interleukin‐4; Tregs, regulatory T cells; VEGF, vascular endothelial growth factor.

Efferocytosis also exhibits complex crosstalk with other key biological processes in the TME. For instance, it is closely associated with cellular senescence. Therapy‐induced senescent cells actively evade phagocytic clearance by macrophages by upregulating “don't‐eat‐me” signals such as CD47 and CD24 [16, 44, 152]. These uncleared senescent cells continuously secrete the senescence‐associated secretory phenotype (SASP), exacerbating chronic inflammation and tissue damage, thereby promoting tumor recurrence and therapy resistance [153] (Figure 5).

## 6 Therapeutic Strategies Targeting Efferocytosis Pathways

Based on an in‐depth understanding of the mechanisms governing efferocytosis, a context‐dependent bidirectional regulatory paradigm has emerged for its therapeutic implications in human diseases. In chronic inflammatory disorders, attenuated or abrogated efferocytic function is a pivotal driver of pathological progression, directly leading to the accumulation of ACs, the initiation of secondary necrosis, and the perpetuation of relentless inflammatory storms. Conversely, within the TME, this intrinsic clearance process is hijacked by malignant cells to establish immunosuppressive barriers and promote immune evasion, marked by M2 macrophage polarization and PD‐L1 upregulation.

Thus, future therapeutic paradigms must adhere to the core precision medicine principle that “context dictates direction” and adopt stratified strategies. For inflammatory diseases, the priority is to potentiate and activate efferocytic pathways; examples include administering SPMs or TAM receptor agonists, which aim to reinitiate the inflammatory resolution program and promote tissue repair. In contrast, for tumor malignancies, targeted inhibition and blockade of efferocytosis‐associated signaling cascades are warranted, such as the use of anti‐CD47 antibodies or MerTK inhibitors, designed to dismantle the immune‐tolerant niche established by tumors. Ultimately, the key to successful therapeutic intervention hinges on the development of tissue‐targeted delivery technologies, which ensure the precise delivery of therapeutic agents to lesion sites, thereby circumventing the therapeutic paradoxes and potential risks associated with systemic modulation of efferocytosis [154].

### 6.1 Boosting Efferocytosis for Inflammation Resolution and Tissue Repair

For most inflammation‐associated disorders, potentiating efferocytosis represents a core therapeutic strategy. This approach not only directly facilitates the resolution of inflammation but also fundamentally reduces the release of DAMPs at the source, serving as a definitive avenue to break the vicious cycle of inflammation. Except for malignancies, this regimen constitutes the predominant therapeutic direction for efferocytosis‐targeted interventions (Table 1).

**TABLE 1:** Efferocytosis‐targeted intervention measures and related preclinical or clinical trial results for inflammation and immune‐related diseases.

Therapeutic mechanism	Intervention method	Target	Molecular mechanism	Significance	Level of evidence
**Exogenous supplementation**	ACs	NA	Supplement exogenous ACs to induce macrophage chemotaxis and enhance efferocytosis.	Attenuate the progression of diseases such as RA [157, 158].	Phase 1/2 clinical trial (NCT02903212)
	Recombinant MFG‐E8	MFG‐E8	Activates the integrin β3/SOCS3/STAT3 signaling pathway and drives the polarization of microglia from the proinflammatory M1 phenotype toward the anti‐inflammatory M2 phenotype.	Significantly attenuates neuroinflammation, cerebral edema, and neuronal apoptosis (*p* < 0.05) [175].	Preclinical animal models
	Recombinant Gas6	Gas6	Activates the Axl receptor, upregulates the expression of its downstream SOCS1 and SOCS3, thereby inhibiting the release of proinflammatory cytokines such as IL‑1β and TNF‑α, and alleviating neuroinflammation following ICH.	Intranasal administration of recombinant Gas6 significantly improved neurological behavioral scores and reduced brain water content in the ipsilateral basal ganglia of mice (*p* < 0.01) [176].	Preclinical animal models
**Regulation of receptors and signaling pathways**	Losmapimod	TIM‐4	The oral p38 inhibitor Losmapimod reduces p38 activity in macrophages from elderly individuals, thereby restoring p300 function, TIM‐4 expression, and efferocytotic capacity.	Oral administration of Losmapimod to elderly volunteers resulted in significantly reduced accumulation of ACs in blisters (*p* < 0.05), decreased LDH levels, restored macrophage TIM‑4 expression (*p* < 0.05), and elevated levels of resolution‑associated factors (*p* < 0.05) [166].	Investigator‐initiated clinical trial
	Anti‐CD47 antibody	CD47	Anti‐CD47 antibodies block the CD47‐SIRPα signaling pathway, suppress downstream SHP1 phosphorylation, restore the efferocytic capacity of macrophages toward ACs, and thereby attenuate atherosclerotic plaque formation.	Treatment significantly reduced plaque area in the aortic sinus and necrotic core size (both *p* < 0.001), and enhanced in vitro efferocytosis efficiency (*p* < 0.01) [85, 87].	Preclinical animal models
	Guanxinkang Decoction	Tyro3, Axl, MerTK, MFG‐E8	Guanxinkang Decoction alleviates atherosclerotic inflammation by enhancing the phagocytic and efferocytic capacity of macrophages through upregulating efferocytosis‑related molecules (AXL, MerTK, Tyro3, MFG‐E8), while inhibiting the MAPKs/NF‑κB signaling pathway by reducing the phosphorylation of p38, JNK, ERK1/2 and NF‑κB p65.	Guanxinkang Decoction significantly reduced serum levels of TC, TG and LDL‐C (all *p* < 0.01), decreased aortic plaque area (*p* < 0.01), downregulated the expression of proinflammatory cytokines IL‐1β, IL‐6 and TNF‐α (all *p* < 0.01), enhanced phagocytic capacity (*p* < 0.05), and upregulated the expression of α‐SMA, a marker of plaque stability (*p* < 0.05) [161].	Preclinical animal models
	Disulfiram	MerTK	Disulfide exerts antiatherosclerotic effects by inhibiting GsdmD‐mediated pyroptosis and IL‐1β secretion, inducing autophagy in a GsdmD‐independent manner, enhancing MerTK‐mediated efferocytosis, and modulating the gut microbiota.	Disulfide significantly reduced atherosclerotic lesion area and necrotic core (*p* < 0.01), inhibited IL‑1β release by approximately 80% (*p* < 0.0001), and enhanced efferocytosis by approximately 2‑fold (*p* < 0.05) [177].	Preclinical animal models
	URMC‐099	MLK3	A MLK3 inhibitor improves the balance of M1/M2 phenotypes, reduces neuroinflammation, promotes the activation of anti‐inflammatory M2 microglia, and further enhances Aβ clearance, synaptic integrity, and hippocampal neurogenesis.	URMC‐099 significantly reduced cortical Aβ burden (*p* < 0.05) and hippocampal Aβ burden (*p* < 0.05) [178].	Preclinical animal models
**Regulation of metabolism and lipid efflux**	GW3965	LXR	GW3965 promotes cholesterol efflux via upregulating the expression of ABCA1 and ABCG1 in macrophages and suppresses NF‑κB‑mediated inflammatory responses.	GW3965 significantly restored sensory nerve conduction velocity in aged mice (*p* < 0.01) and decreased the levels of proinflammatory cytokines TNF‑α and IL‑1β (*p* < 0.05) [179].	Preclinical animal models

Abbreviations: Aβ, amyloid beta; ACs, apoptotic cells; ICH, intracerebral hemorrhage; RA, rheumatoid arthritis.

#### 6.1.1 Positive Regulation of Efferocytosis via Key Receptors and Signaling Pathways

Efferocytosis plays a central role in maintaining tissue homeostasis and resolving inflammation. Strategies to enhance this process focus on modulating phagocytic receptors, supplying exogenous ACs or their signaling molecules, and regulating downstream metabolic pathways. The following approaches highlight promising therapeutic avenues for inflammatory and autoimmune diseases.

A direct strategy to enhance efferocytosis is the administration of ACs or their metabolites. Experimentally, AC‐derived metabolites such as AMP, GMP, and creatine exert anti‐inflammatory and proresolving effects [155]. In animal models of arthritis, administration of ACs via various routes alleviates disease severity [156]. Based on these findings, a Phase I/II clinical trial (NCT02903212) has been proposed to assess autologous AC infusion in refractory RA [157, 158]. Moreover, PS, the dominant “eat‐me” signal, can be delivered via liposomal formulations. In atherosclerotic models, PS‐liposomes promote plaque regression by enhancing macrophage efferocytosis [159]. Apoptotic bodies derived from M2 macrophages (M2‐ApoBDs) further contribute to immune tolerance by reprogramming phagocytes toward a regulatory phenotype, as demonstrated in SLE models, where they drive Treg differentiation and suppress disease progression [160].

Enhancing the activity of efferocytosis receptors and their associated bridging molecules represents a key strategy to improve phagocytic clearance capacity. Among these, the MerTK signaling pathway can be potentiated by small molecules or natural compounds. For instance, Guanxinkang Decoction (GXK) reduces plaque burden and inflammation in atherosclerosis models by upregulating the expression of MerTK and MFG‐E8 [161]. Similarly, hydroxychloroquine, a therapeutic agent for SLE, enhances the expression of MerTK and its ligand, Gas6, in macrophages while inhibiting the production of inflammatory cytokines such as IL‐6 [162]. Furthermore, bridging molecules, including MFG‐E8 and Gas6, facilitate the recognition of PS. Recombinant MFG‐E8 ameliorates tissue fibrosis in systemic sclerosis models [163], whereas glucocorticoids, which are widely used for autoimmune diseases, exert their therapeutic effects partially by upregulating the expression of efferocytosis mediators such as MFG‐E8, Protein S, and annexin A1 [61, 164, 165]. Furthermore, TIM‐4 serves as another critical receptor. In senescent phagocytes, its expression is negatively regulated by the aberrantly activated p38 MAPK pathway. This TIM‐4 deficiency renders macrophages incapable of efficiently recognizing and phagocytosing local ACs, thereby resulting in the accumulation of cellular debris and the persistence of inflammation. Notably, oral administration of a p38 MAPK inhibitor (e.g., Losmapimod) can successfully alleviate inhibition of the relevant pathway, rescuing and re‐upregulating TIM‐4 expression on the surface of macrophages derived from elderly individuals [166].

The LXR and PPARγ pathways integrate efferocytosis with anti‐inflammatory metabolic responses. Activation of these receptors enhances cholesterol efflux and upregulates efferocytosis receptors such as MerTK and CD36. In lupus models, gut microbiota‐derived metabolites activate PPARγ/LXR in macrophages, boosting CD36‐dependent clearance of ACs and attenuating disease. This highlights the role of metabolic rewiring in resolving inflammation postefferocytosis [167].

#### 6.1.2 Inhibiting the “Don't‐Eat‐Me” Signals

In various pathological models, CD47, a key “don't‐eat‐me” signal, is frequently overexpressed [86, 168]. By binding to SIRPα on the macrophage surface and activating the downstream SHP1 phosphorylation pathway, CD47 suppresses macrophage efferocytosis, leading to the accumulation of ACs in tissues, expansion of necrotic areas, and sustained inflammatory responses, thereby driving disease progression. Studies have confirmed that blocking CD47 significantly alleviates inflammation and suppresses pathological development.

Taking atherosclerosis as an example, therapeutic strategies employing anti‐CD47 monoclonal antibodies or SIRPα‐Fc fusion proteins have demonstrated efficacy. The former directly binds to CD47, blocking its interaction with SIRPα, while the latter acts as a soluble decoy receptor that competitively occupies CD47. Both approaches effectively restore macrophage efferocytosis. Animal experiments indicate that such treatments markedly enhance the clearance of ACs by macrophages, reduce the necrotic core area within plaques, and promote polarization toward the M2 anti‐inflammatory macrophage phenotype [85, 87]. As a result, these interventions improve plaque stability and offer a novel immunotherapeutic strategy for related diseases.

#### 6.1.3 Supplementation With SPMs Enhances Efferocytosis

SPMs are endogenous small molecules primarily synthesized by structural and immune system cells from dietary ω‐3 PUFAs [169]. As key regulators of inflammatory resolution, SPMs differ from traditional anti‐inflammatory drugs in that they do not inhibit immune responses; instead, they actively promote inflammation resolution and tissue repair. Research has demonstrated that SPMs upregulate the expression of efferocytosis‐associated receptors (MerTK) on macrophage surfaces and simultaneously facilitate M2 polarization, thereby exerting anti‐inflammatory effects. In multiple inflammatory disease models, researchers have found that SPM supplementation yields significant disease amelioration, confirming its potent therapeutic efficacy [170, 171, 172].

#### 6.1.4 Exploration of New Technologies and Therapies

With the continued advancement of nanoparticle‐based drug delivery systems, the development of nanomedicines targeting efferocytosis‐associated signaling pathways has emerged as a novel and highly efficient therapeutic strategy. Yuan et al. designed and fabricated an efferocytosis‐mimicking nanovesicle (EMNV, namely, S1P‐PS‐MMV@SPD). By integrating three key efferocytic signals, including S1P (the “find‐me” signal) for recruiting phagocytes, PS (the “eat‐me” signal) for facilitating phagocytic recognition, and SPD (the apoptotic metabolite) for inducing immunomodulation, this nanovesicle achieves precise targeting of inflamed joints and the spleen. In a murine model of RA, the nanovesicle exerted remarkable anti‐inflammatory effects and effectively alleviated joint swelling and bone erosion [173]. Besides, Tan et al. engineered macrophages that enhance efferocytosis through a triple synergistic mechanism: overexpressing CCR2 to guide the precise migration of macrophages to myocardial injury sites; overexpressing cleavage‐resistant MerTK to prevent receptor inactivation by ADAM17 and thus sustain efferocytic function; and simultaneously loading ROS‐responsive PEP‐20 (a CD47 antagonist) to specifically block the “don't‐eat‐me” signal in the high‐ROS microenvironment of injured tissues, thereby achieving efficient clearance of ACs. This approach markedly reduces residual ACs in the myocardium, inhibits proinflammatory cytokines, promotes the production of anti‐inflammatory cytokines and SPMs, alleviates inflammatory infiltration, accelerates the resolution of active inflammation, and ultimately improves cardiac function. Collectively, it provides a highly efficient therapeutic strategy for myocardial ischemia–reperfusion injury [174]. These advanced therapeutic strategies represent an innovative immunomodulatory therapy that achieves dual effects: suppressing inflammatory initiation and promoting active inflammation resolution via the precise targeting and restoration of local efferocytosis, thereby markedly improving disease prognosis.

### 6.2 Inhibiting Efferocytosis in the Tumor Microenvironment

Aberrant activation of efferocytosis within the TME drives malignant progression through diverse mechanisms. TAMs, by excessively engulfing ACs, release prosurvival factors that enhance tumor cell viability while fostering an immunosuppressive milieu that compromises antitumor immunity. This pathological reprogramming of efferocytosis has emerged as a promising therapeutic target. Emerging evidence suggests that inhibiting key efferocytosis receptors (such as MerTK) or neutralizing “eat‐me” signals effectively reverses tumor‐associated immunosuppression. Notably, preclinical models have demonstrated that combining efferocytosis‐targeted agents with immune checkpoint inhibitors synergistically suppresses tumor growth and prolongs survival. This chapter will elucidate current therapeutic strategies targeting dysregulated efferocytosis, evaluate their translational potential, and provide insights into novel combinatorial approaches for cancer treatment (Table 2).

**TABLE 2:** Efferocytosis‐targeted intervention measures and related preclinical or clinical trial results for cancer.

Therapeutic mechanism	Intervention method	Target	Molecular mechanism	Significance	Level of evidence
**Targeting “eat‐me” signals**	Bavituximab	PS	By binding to PS, bavituximab blocks the interaction of PS with immune cell receptors and reverses PS‐mediated immunosuppression. This promotes the conversion of macrophages from the immunosuppressive M2 phenotype to the activated M1 phenotype, thereby enhancing the cytotoxic activity of immune cells against tumor cells.	In patients with newly diagnosed IDH‑wild‐type glioblastoma, standard chemoradiotherapy combined with bavituximab met the primary endpoint, with a 12‑month OS rate of 72.7%. The median PFS was 6.9 months and the median OS was 15.4 months. Mechanistic studies demonstrated that the number of immunosuppressive MDSCs in tumor specimens was significantly reduced after treatment (*p* < 0.05) [181].	Phase 2 clinical trial (NCT03139916)
	Bavituximab	PS	Same as above	In patients with treatment‐naïve unresectable hepatocellular carcinoma, bavituximab combined with pembrolizumab achieved the primary endpoint, with a confirmed ORR of 32.1% (9/28); the median PFS was 6.3 months [210].	Phase 2 clinical trial (NCT03519997)
	Bavituximab	PS	Same as above	Among patients with previously treated advanced non–small‐cell lung cancer, no significant differences were observed in OS and PFS between the docetaxel plus bavituximab group and the placebo group. However, in patients who received subsequent immune checkpoint inhibitor therapy, OS was significantly prolonged in the bavituximab group compared with the placebo group (*p* = 0.006) [211].	Phase 3 clinical trial (NCT01999673)
	BPRDP056	PS	This drug specifically binds to exposed PS via its humanized anti‑phosphatidylserine antibody. Following cellular internalization through endocytosis, it releases its cytotoxic payload (a tubulin inhibitor), thereby inducing tumor cell apoptosis and inhibiting tumor angiogenesis.	In multiple human tumor xenograft mouse models, BPRDP056 significantly suppressed tumor growth and achieved complete remission in several models, with tumor volume inhibition rates exceeding 90% (*p* < 0.001). Furthermore, no obvious body weight loss or systemic toxicity was observed at high doses [212].	Preclinical animal models
**Targeting the TAM receptor family**	Sitravatinib	Tyro3, Axl, MerTK	Sitravatinib is a multitargeted receptor tyrosine kinase inhibitor that potently suppresses the TAM family (Tyro3, Axl, MerTK) as well as VEGFR2. It directly inhibits tumor growth while promoting the conversion of M2 macrophages to the M1 phenotype, thereby remodeling the immunosuppressive tumor microenvironment.	In the advanced hepatocellular carcinoma cohort, the combination therapy group exhibited an ORR of 20.0%, a DCR of 73.3%, a mPFS of 5.5 months, and a median mOS of 14.8 months. In the gastric cancer/gastroesophageal junction cancer cohort, the combination therapy group achieved an ORR of 21.4%, a DCR of 64.3%, an mPFS of 4.1 months, and an mOS of 9.2 months [213].	Phase 1b/2 clinical trial (NCT03941873)
	Bemcentinib	Axl	Bemcentinib inhibits AXL kinase activity and blocks downstream signaling pathways (PI3K/AKT, MAPK/ERK), thereby suppressing tumor cell growth, inducing apoptosis, and reversing resistance to immunotherapy in tumor cells.	In patients with advanced lung cancer, the addition of bemcentinib enhances the response rate to anti‐PD‐1 therapy [214].	Phase 2 clinical trial (NCT03184571)
	MRX‐2843	MerTK	MRX‐2843 blocks MerTK signaling and attenuates macrophage phagocytosis, promoting the recognition of antigens and danger signals released from apoptotic tumor cells by immune cells, and consequently activating both innate and adaptive immune responses.	In a mouse model of acute myeloid leukemia, MRX‐2843‐mediated inhibition of efferocytosis significantly prolonged survival (median survival was extended from approximately 25 days in the control group to more than 40 days in the treatment group, *p* < 0.001), and markedly reduced tumor burden and the proportion of immunosuppressive macrophages [215].	Preclinical animal models
	ONO‐7475	Axl	ONO‐7475, an AXL inhibitor (in combination with osimertinib and an FGFR inhibitor), reverses acquired resistance to osimertinib in EGFR‐mutant non–small‐cell lung cancer and restores tumor cell sensitivity to treatment by specifically inhibiting AXL phosphorylation and the activation of its downstream PI3K/AKT and MAPK signaling pathways.	In xenograft mouse models harboring EGFR mutations with secondary resistance, the triple‐combination regimen containing ONO‐7475 significantly suppressed tumor growth compared with monotherapy or dual‐agent therapy (*p* < 0.01) and induced more durable tumor regression [192].	Preclinical animal models
	BMS794833	MerTK	This agent employs a polypharmacological strategy to simultaneously block key signaling cascades during TAM polarization, suppress the expression of protumorigenic genes, and upregulate proinflammatory cytokines, thereby reprogramming protumorigenic M2‑like TAMs into an inflammatory phenotype with potent anti‑tumor activity.	In mouse models of triple‐negative breast cancer, treatment with BMS‑794833 significantly suppressed tumor growth (*p* < 0.001) and reduced the number of pulmonary metastatic foci (*p* < 0.05) [216].	Preclinical animal models
**Targeting “don't‐eat‐me” signals**	Evorpacept	CD47	Evorpacept binds to CD47 with high affinity, blocks the CD47–SIRPα interaction, abrogates the immune evasion mechanism of tumor cells, and enables macrophages to recognize and phagocytose tumor cells.	In patients with head and neck squamous cell carcinoma, combination therapy with pembrolizumab achieved an ORR of 20.0%. In patients with HER2‑positive gastric cancer/gastroesophageal junction cancer, combination therapy with trastuzumab resulted in an ORR of 21.1%, and the regimen exhibited an acceptable safety profile [217].	Phase 1 clinical trial (NCT03013218)
**Targeting metabolic reprogramming**	PY314	TREM2	By inhibiting TREM2, PY314 disrupts fatty acid uptake and metabolism in macrophages and reduces lipid droplet formation, thereby attenuating their protumor immunosuppressive functions.	PY314 plus pembrolizumab demonstrated limited antitumor activity in patients with advanced renal cell carcinoma refractory to prior immune checkpoint inhibitor therapy. Among the 17 treated patients, only one achieved a partial response (PR, 5.9%), four had stable disease (SD, 23.5%), and the mPFS was 1.4 months [207].	Phase 1 clinical trial (NCT04691375)
	CB‐1158	Arg1	CB‐1158 inhibits the activity of Arg1, thereby blocking myeloid cell‐mediated depletion of L‐arginine. This mechanism restores the proliferative capacity of T cells and NK cells, and drives the tumor microenvironment to shift toward a proinflammatory immune phenotype.	Both CB‐1158 monotherapy and its combination with immunotherapy resulted in significant inhibition of tumor growth (*p* < 0.05). Meanwhile, the treatments markedly increased the infiltration of CD8+ T cells and NK cells, while significantly reducing the proportion of myeloid cells (*p* < 0.05) [199].	Preclinical animal models
	OATD‐02	Arg1/2	OATD‐02 is a novel dual arginase (ARG1/ARG2) inhibitor. By effectively inhibiting both extracellular and intracellular arginases, it restores the availability of L‐arginine in the tumor microenvironment and reduces polyamine levels, thereby relieving metabolic constraints on T cells and potentiating antitumor immune responses.	In the CT26 murine tumor model, combination treatment of OATD‐02 with anti‑PD‑1 therapy significantly prolonged median survival to 41.5 days (vs. 29.5–32 days for monotherapy, *p* < 0.05), and markedly elevated tumor‑infiltrating T cells (*p* < 0.05) as well as the expression of the T‐cell activation marker CD69 (*p* < 0.0001).	Preclinical animal models Phase 1 clinical trial (NCT05759923)

Abbreviations: Arg1/2, arginase 1/2; MDSCs, myeloid‐derived suppressor cells; mOS, median overall survival; mPFS, median progression‐free survival; ORR, objective response rate; OS, overall survival; PD‐1/PD‐L1, programmed cell death protein 1/ligand 1; PFS, progression‐free survival; PR, partial response; PS, phosphatidylserine; SD, stable disease.

#### 6.2.1 Targeting Efferocytosis‐Associated Receptors and Signaling Pathways

PS, a pivotal “eat‐me” signal in efferocytosis, plays a central role in tumor immune evasion. Its targeted agent, bavituximab, exerts antitumor effects through a dual mechanism: on the one hand, it binds to the abnormally exposed PS on the surface of tumor vascular endothelial cells and apoptotic cancer cells, activates macrophage‐mediated antibody‐dependent cellular cytotoxicity (ADCC), and induces tumor vascular occlusion, ischemia, and necrosis [180]; on the other hand, it blocks PS‐mediated immunosuppressive signals, promotes the secretion of proinflammatory cytokines such as TNF‐α, eliminates myeloid‐derived suppressor cells (MDSCs), induces M1 macrophage polarization and DC maturation, thereby activating the antitumor immune response of cytotoxic T lymphocytes (CTLs) [180, 181]. This agent can synergize with chemotherapy or radiotherapy to enhance therapeutic efficacy, and its specific targeting of tumor‐associated PS significantly reduces toxicity to normal tissues [182, 183, 184]. Currently, multiple clinical trials are integrating nanoparticle delivery systems and gene editing technologies to further explore their potential.

Meanwhile, the TAM receptor family (Tyro3, Axl, MerTK), as core regulatory molecules of efferocytosis, has emerged as an important target for TME remodeling and immune evasion [185, 186]. Among these receptors, MerTK inhibition can block the ERK/SP1/SLC7A11 pathway mediated by MerTK, suppress ferroptosis, and reverse resistance to PD‐L1/PD‐1 inhibitors [187]. Combined treatment with MerTK inhibitors can also activate the STING/IFN pathway to ameliorate the TME [187]. Axl inhibition enhances DC activation and CD8^+^ T‐cell infiltration by suppressing epithelial–mesenchymal transition (EMT) and PD‐L1 expression [188, 189]. Moreover, small‐molecule inhibitors (bemcentinib, TP‐0903) and antibody–drug conjugates (BA3011) have entered clinical validation [190]. In non–small‐cell lung cancer, combination therapy with the Axl inhibitor ONO‐7475 and osimertinib can overcome drug resistance [191], while the triple regimen of osimertinib, ONO‐7475, and an FGFR inhibitor achieves even greater efficacy [191, 192]. In addition, Tyro3 inhibition can significantly enhance the therapeutic efficacy of anti‐PD‐1/PD‐L1 therapy and improve the immunosuppressive microenvironment [193].

However, the potential side effects of TAM receptor‐targeting drugs, notably on retinal function, warrant careful consideration [194, 195]. These issues must be addressed with urgency. In summary, targeting the TAM receptor family as an antitumor strategy demonstrates significant potential, not only by directly suppressing tumor proliferation and metastasis but also by modulating the TME, thus warranting further in‐depth investigation.

#### 6.2.2 Targeting the “Don't‐Eat‐Me” Signals

As mentioned earlier, senescent tumor cells can evade phagocytosis mediated by macrophages by expressing “don't‐eat‐me” signals (CD47 and CD24). During tumor treatment, inducing a senescent phenotype is a common phenomenon, which is also one of the important reasons for tumor recurrence [196]. Therefore, how to effectively eliminate senescent tumor cells has become a key scientific issue in current research. In vitro experiments by Biedermann et al. demonstrated that anti‐CD47 antibodies (such as B6H12) could effectively block the “don't‐eat‐me” signal and significantly enhance the phagocytic clearance ability of macrophages against tumor cells. Besides, in animal models, the combined use of anti‐CD47 antibodies and tafasitamab could significantly reduce tumor volume in mice and significantly prolong their survival period [197]. Yu et al. discovered a potential antiphagocytic signaling molecule, GSTK1. By blocking it with siRNA‐GSTK1 nanoparticles, they significantly enhanced the phagocytic efficiency of macrophages toward cancer cells and effectively compensated for the insufficient efficacy of anti‐CD47 antibodies [198]. Overall, CD47 has gradually emerged as a new target for immunotherapy.

A key consideration pertains to the optimal stage of tumor progression for interrupting “don't‐eat‐me” signaling. It is well established that macrophages, upon engulfing apoptotic tumor cells, contribute to an immunosuppressive environment that drives tumor progression. Poorly timed intervention thus risks counterproductive outcomes that may inadvertently accelerate disease. Similarly, the decision to block “eat‐me” signals presents a related paradox: such blockade may suppress macrophage phagocytosis of tumor cells and consequently impair antigen presentation. These problems constitute the core contradictions in treatment and urgently require in‐depth research and discussion.

#### 6.2.3 Regulation of Metabolic Pathways

Targeting the metabolic mechanisms underlying efferocytosis has emerged as a vital strategy for tumor immunotherapy, with considerable potential, particularly for modulating macrophage metabolism. Arginine metabolism is a key process that drives the immunosuppressive microenvironment, and inhibition of Arg1, its core enzyme, can restore T‐cell activity and enhance antitumor immunity. In pancreatic cancer models, myeloid cell‐specific Arg1 knockout significantly reduces liver metastasis and increases CD8^+^ T‐cell infiltration. The small‐molecule inhibitor CB‐1158 exerts selective blockade of Arg1 activity, effectively abrogating the immunosuppressive function of MDSCs, promoting CD8^+^ T‐cell activation, and generating synergistic antitumor effects in combination with immune checkpoint inhibitors across multiple solid tumors [199]. Another dual‐target inhibitor of Arg1/Arg2, OATD‐02, reduces the number of tumor‐infiltrating myeloid cells and, when combined with anti‐PD‐1 antibodies, achieves marked suppression of tumor growth [200, 201]. Furthermore, vaccines based on Arg1 peptides eliminate immunosuppressive MDSCs and M2‐type macrophages by activating antigen‐specific T cells. Preclinical studies have demonstrated that their combination with anti‐PD‐1 antibodies can reprogram the TME, and such vaccines are currently undergoing Phase I clinical trials [202, 203].

Downstream of arginine metabolism, arginine is catabolized to ornithine by arginase, which is then converted to putrescine via ODC. This polyamine synthesis pathway exacerbates immunosuppression. Inhibition of ODC (e.g., with DFMO) reduces polyamine accumulation, reverses the immunosuppressive phenotype of TAMs, and facilitates T‐cell infiltration. In glioblastoma models, DFMO significantly prolongs the survival of tumor‐bearing mice by reducing the intracellular acidity of TAMs [204]. Meanwhile, TREM2 serves as a critical regulator of TAM lipid metabolism, inducing an immunosuppressive phenotype via recognition of ligands such as lipoproteins [60, 205]. PY314, a monoclonal antibody targeting TREM2, can specifically block its signaling pathway, deplete immunosuppressive TAMs in the TME, and shift the microenvironment from an immunosuppressive to an inflammatory state [206]. Preclinical studies have shown that PY314 monotherapy exhibits a manageable safety profile and potential for disease stabilization in platinum‐resistant ovarian cancer. A Phase I clinical trial combining PY314 with pembrolizumab is currently underway to further validate its efficacy [207].

#### 6.2.4 Exploration of New Technologies and Therapies

Meanwhile, several emerging treatment strategies, such as new nanomedicines, warrant further investigation. These drugs have demonstrated excellent effects in both in vitro and in vivo experiments and have the potential for further clinical research. Liu et al. developed a copper‐tetrahydroxybenzoquinone (Cu‐THBQ) metal–organic framework (MOF)‐based nanovaccine (Cu‐THBQ/AX) capable of precisely releasing the ERK5 inhibitor XMD8‐92. This nanovaccine inhibits ERK5 phosphorylation in macrophages, thereby downregulating MerTK receptor expression and blocking its recognition of PS exposed on the surface of ACs. Consequently, apoptotic tumor cells evade effective phagocytosis and clearance by TAMs, transitioning instead into a proinflammatory secondary necrotic state. This mechanism disrupts efferocytosis‐mediated immune suppression while amplifying antigen exposure and inflammatory signaling within the TME [208]. In addition, Zhou et al. successfully suppressed TAM efferocytosis by delivering MerTK‐targeted small interfering RNA (siMerTK) via lipid nanoparticles (LNPs), thereby reversing the immunosuppressive TME. This approach synergized with PD‐1 blockade to significantly enhance antitumor immunity, offering a novel combination therapeutic strategy for metastatic colorectal cancer. The study highlights the potential of organ‐specific MerTK silencing using LNP‐based RNA interference to disrupt tumor immune evasion mechanisms and amplify checkpoint inhibitor efficacy [209].

### 6.3 Challenges and Future Perspectives

Drug therapies targeting efferocytosis are confronted with multiple core challenges, primarily stemming from the duality and complexity of its functions. As previously described, impaired efferocytic function in inflammatory diseases requires augmentation to facilitate the resolution of inflammation. In contrast, within the context of cancer, tumors exploit efferocytosis to construct an immunosuppressive milieu, thereby warranting blockade of the associated signaling pathways. This dichotomy renders it challenging to unify therapeutic strategies, especially in patients with comorbidities such as cancer complicated by SLE. Besides, targeting specificity poses a notable challenge. Efferocytosis is ubiquitous across tissues, yet receptor expression and AC burden display marked tissue specificity, complicating precise targeting within disease‐affected sites. Therapeutic agents are also associated with notable side effects; for instance, CD47 inhibitors may disrupt erythrocyte function and induce anemia [218]. Moreover, disease heterogeneity, including tumor molecular subtype diversity and the complexity of the TME, makes treatment responses difficult to predict, and reliable biomarkers for evaluating therapeutic efficacy remain lacking. Furthermore, efferocytosis involves the coordination of multiple sequential steps (recognition, internalization, and degradation) and signaling pathways, making interventions targeting a single molecular target prone to failure. Compounding these challenges, age‐related functional decline in phagocytes (polarization toward an efferocytosis‐deficient phenotype) further complicates therapeutic intervention in elderly patients. Collectively, these factors severely impede the efficiency of clinical translation for efferocytosis‐targeted therapies.

Future research should focus on developing precision‐targeting strategies to overcome the aforementioned challenges. The core directions include: comprehensively dissecting the molecular mechanisms of efferocytosis across distinct tissues, disease stages and age contexts; leveraging interdisciplinary approaches (integrating single‐cell sequencing, metabolomics and AI modeling) to identify disease‐specific receptor signaling pathways (the dynamic expression of MerTK within the TME); and engineering highly selective therapeutics (such as nanoparticle‐based drug delivery systems) to minimize off‐target effects. Concurrently, it is imperative to establish a functional efferocytosis assessment system and develop novel biomarkers (e.g., tissue‐specific apoptotic signaling molecules in serum) for real‐time monitoring of treatment responses, and to explore synergistic mechanisms with immune checkpoint inhibitors (e.g., combining MerTK inhibitors with PD‐L1 antibodies). For age‐related efferocytic decline, age‐stratified intervention strategies need to be formulated. The goal is to achieve a “disease–target–patient” triad of precision medicine, thereby providing a novel and safe therapeutic paradigm of efferocytosis‐targeted therapy for inflammatory disorders and malignancies.

## 7 Conclusions and Future Directions

Efferocytosis is a core physiological process that maintains tissue homeostasis. Mechanistic investigations into this pathway have reshaped our understanding of inflammation resolution, immune tolerance, and tissue repair. This review systematically summarizes the molecular mechanisms underlying efferocytosis and related metabolic reprogramming, as well as its pathological roles in aging, chronic inflammation, autoimmunity, and fibrosis. Emphasis is placed on its context‐dependent dual functions in disease development. The paradigm‐shifting finding that tumor cells can hijack efferocytosis to establish immunosuppressive microenvironments challenges the long‐standing concept that this process is uniformly protective. Rather than being uniformly beneficial or harmful, efferocytosis acts as a dynamic regulator that requires disease‐tailored modulation.

This framework supports a context‐dependent therapeutic strategy: enhancing efferocytosis in chronic inflammatory diseases to promote resolution and repair, while inhibiting it in cancer to facilitate immune‐mediated tumor clearance. Such an approach embodies precision medicine by moving beyond uniform treatment paradigms. Major challenges remain for clinical application, including precise assessment of efferocytosis status in disease, targeted tissue delivery, and avoidance of adverse effects. Future directions should prioritize biomarker‐driven patient stratification and the development of targeted delivery platforms to enable safe, context‐specific modulation of efferocytosis.

From a broader perspective, efferocytosis research has unveiled an intricate regulatory network integrating immunity, metabolism, and tissue repair. Its metabolic reprogramming effects, including dynamic modulation of amino acid, lipid, and glucose metabolism, highlight the profound interplay between immune cell function and metabolic states, offering novel insights into immunometabolic disease mechanisms. Notably, efferocytosis intersects with emerging fields such as cellular senescence, mechanosensing, and epigenetic regulation, creating interdisciplinary opportunities for exploring disease mechanisms. Importantly, investigating age‐related efferocytosis dynamics within the “aging–inflammation–fibrosis” axis may reveal novel strategies to combat age‐associated pathologies.

Future research on efferocytosis will focus on several interrelated directions. Advances in precise functional evaluation techniques, including single‐cell multiomics analysis and in vivo imaging, will facilitate the mapping of dynamic efferocytic landscapes across different stages of disease. Meanwhile, elucidating the crosstalk mechanisms between efferocytosis and key physiological processes in tumor immunology, neurodegenerative diseases and cardiovascular diseases is critical for understanding its functions under pathological conditions. At the translational medicine level, the development of disease‐specific modulators targeting TAM receptors will accelerate the clinical translation of efferocytosis‐targeted therapies. Notably, integrating efferocytosis‐associated monitoring indicators into clinical trial design can serve as valuable biomarkers for assessing therapeutic response and prognosis. With deeper insights into the mechanisms underlying efferocytosis, precise intervention strategies tailored to the individual biological characteristics of patients are expected to be achieved, thereby promoting the advancement of context‐driven personalized medicine.

## Author Contributions

Lei Wang and Jingjing Ge contributed equally to the conceptualization, data analysis, and manuscript drafting. Ruyue Zhang and Zehua Wang assisted in data collection and technical support. Yihua Fang coordinated the research process. Yongxu Jia, Ruyue Zhang, and Yanru Qin supervised the study and revised the manuscript. All the authors approved the final version.

## Funding

This work was supported by the National Natural Science Foundation of China (No. 82273381), the Henan Joint Co‐construction Project for Medical Science and Technology Research (No. LHGJ20240222), the Natural Science Foundation of Henan Province (Young Scientists Fund, No. 252300423914), and the Natural Science Foundation of Henan Province (Young Students’ Science Fund Project, No. 262300422728).

## Ethics Statement

The authors have nothing to report.

## Conflicts of Interest

The authors declare no conflicts of interest.

## Data Availability

The authors have nothing to report.
